# Subgingival Microbiota and Cytokines Profile Changes in Patients with Periodontitis: A Pilot Study Comparing Healthy and Diseased Sites in the Same Oral Cavities

**DOI:** 10.3390/microorganisms9112364

**Published:** 2021-11-16

**Authors:** Pauline Esparbès, Arnaud Legrand, Octave Nadile Bandiaky, Marjorie Chéraud-Carpentier, Hamida Martin, Emmanuel Montassier, Assem Soueidan

**Affiliations:** 1 Department of Periodontology, Faculty of Dental Surgery, UIC 11, Rmes U1229, CHU de Nantes, 44000 Nantes, France; esparbes.pauline@orange.fr (P.E.); assem.soueidan@univ-nantes.fr (A.S.); 2 National Institutes of Health and Medical Research, CIC 1413, CHU de Nantes, 44000 Nantes, France; arnaud.legrand@chu-nantes.fr; 3 Division of Fixed Prosthodontics, University of Nantes, 1 Place Alexis Ricordeau, 44042 Nantes, France; octave.bandiaky@chu-nantes.fr; 4 UIC Odontology, CHU, 44000 Nantes, France; marjorie.cheraudcarpentier@chu-nantes.fr (M.C.-C.); hamida.martin@chu-nantes.fr (H.M.); 5 Clinical and Experimental Therapeutics of Infections, Faculty of Medicine, EA 3826, University of Nantes, 44000 Nantes, France; 6 Emergency Department, Nantes University Hospital, 44000 Nantes, France

**Keywords:** subgingival microbiota, periodontitis, dysbiosis, gingival crevicular fluid, 16S rRNA gene sequencing

## Abstract

Periodontitis is a common condition characterized by an exacerbated pro-inflammatory response, which leads to tissue destruction and, ultimately, alveolar bone loss. In this pilot study, we assess the microbiota composition and cytokine profile changes in patients with stage III/IV, grade B/C periodontitis, specifically by comparing healthy and diseased sites in the same oral cavity. Overall, we found that microbiota architecture was significantly disrupted between diseased and healthy sites, and that the clustering was driven, in part, by the increased relative abundances of Synergistetes in diseased sites, as well as the increased abundances of Firmicutes in healthy sites. We also observed that diseased sites were enriched in Synergistetes, TM7, SR1, Spirochaetes, Bacteroidetes and Fusobacteria, and depleted in Firmicutes, Proteobacteria, Tenericutes and Actinobacteria compared to healthy sites. We found that Interleukin-1b, Interleukin-4, Interleukin-10, and Interleukin-17A were significantly overexpressed in diseased sites, whereas Interleukin-6 and TNF-alpha do not differ significantly between healthy and diseased sites. Here, we observed concomitant changes in the subgingival plaque microbiota and cytokines profile, suggesting that this combined alteration could contribute to the pathobiology of periodontitis.

## 1. Introduction

Periodontitis is a common condition for which the consequences gradually increase with age [1]. This chronic inflammation of tooth-supporting tissues is characterized by an exacerbated pro-inflammatory response, ultimately leading to tooth loss. Even though a detailed pathogenesis remains elusive, it is well accepted that bone destruction occurs in response to oral microbiota dysbiosis [2].

A new classification was proposed following the Chicago consensus conference in 2017, co-presented by the American Academy of Periodontology (AAP) and the European Federation of Periodontology (EFP). This new classification replaced the Armitage classification [3,4]. In the new classification scheme, the distinction between aggressive periodontitis and chronic periodontitis disappears in favor of its classification by stage and grade, determined by the severity of bone loss, the speed of progression and the associated risk factors. Necrotizing periodontal disease, periodontal abscesses and endodontic-periodontal lesions are thus considered as separate entities. Periodontitis can be of stage I, II, III or IV, depending on the severity, complexity and distribution of damage. The grade can range from A to C, depending on the history of the disease, its risk of progression and the patient’s personal risk factors [5,6].

Although dysregulated cytokine expression has been linked to periodontal inflammation [2], and dysbiotic oral microbiota to periodontal disease [7,8], few studies investigated interactions between oral microbiota and cytokines in individuals with or without periodontitis [7,9], and no studies have compared healthy and diseased sites in the same oral cavity. Yet, a more developed understanding of functional links between the microbiota community and host cytokine response, based on intra-individual comparison, may help to identify new therapeutic strategies based on oral microbiota modulation.

This study aimed to assess microbiota composition and cytokine profile changes in patients with stage III/IV, grade B/C periodontitis, by comparing healthy and diseased sites in the same oral cavity.

## 2. Materials and Methods

### 2.1. Study Population

This pilot cross-sectional comparative study was carried out according to the Declaration of Helsinki as revised in 2000 for studies involving human participants and was approved by the Ethical Committee at the Nantes University Dental Hospital. The trial protocol was registered in the US Clinical Trials Registry NCT04251650, and all participants were informed of associated risks and benefits and provided their informed consent. These participants were recruited from the Department of Periodontology of the Dental Care Center of Nantes University Hospital between May 2019 and November 2019. Patients were subjects with periodontitis, diagnosed according to the clinical and radiographic criteria proposed by the 2018 International Workshop for the Classification of Periodontal Diseases and Conditions [5,6].

### 2.2. Inclusion/Exclusion Criteria

The inclusion criteria involved smoking and non-smoking patients who speak and understand French; who were over 18 years old; with good general health and at least 12 teeth on the arch (excluding third molars); presented a generalized periodontitis stage III/IV, grade B/C according to the new classification [10]; required non-surgical periodontal treatment, and who provided oral and written consent. The exclusion criteria excluded participants with acute oral lesions; endo-periodontal lesions; ulcero-necrotic gingivitis or periodontitis; a chronic or systemic pathology or treatment that may influence the periodontal microbiota and the response to treatment (immunotherapy, corticotherapy, biotherapy, unbalanced diabetes (glycated hemoglobin [HbA1c] ≥ 7%); acute inflammatory rheumatism; neurological deficiency); subjects using a systemic or local antibiotics in the last 3 months and pregnant and lactating women.

### 2.3. Clinical Examination

A single qualified and experienced operator (P.E.) measured clinical parameters and carried out the samples collection. All patients were interviewed to obtain their demographic data and medical history. All of them benefited from a full-mouth periodontal examination, including their pocket depth (PD, distance from the free gingival margin to the bottom of the pocket), clinical attachment level (CAL, distance from the cemento-enamel junction to the bottom of the pock), bleeding index (FMBS) and plaque score (FMPS). These clinical parameters were assessed at six sites around each tooth (mesiobuccal, midbuccal, distobuccal, mesiolingual, midlingual and distolingual locations) with a millimeter periodontal probe (Colorvue, HuFriedyGroup, Frankfurt am Main, Germany). Additionally, a radiographic examination was performed to consider the alveolar bone loss. All periodontal chart measurements were noted in a programmed Excel table (Microsoft^®^, Redmond, WA, USA, 2011). This made it easier to identify healthy and diseased sites, thus facilitating their selection for gingival fluid and bacterial plaque sampling. Clinical indices, periodontal scores and diagnosis were automatically calculated for each subject.

### 2.4. Gingival Crevicular Fluid (GCF) and Subgingival Plaque (SP) Samples Collection

For each patient, after clinical examination, 3 healthy (PD ≤ 3 mm, without inflammation) and 3 diseased (PD ≥ 5 mm, without BOP) sites were selected. The choice of healthy sites was based on a probing depth of <3 mm and ease of access (usually maxillary and mandibular incisors and canines). For the affected sites, site selection was based on pocket depth >5 mm and ease of access to the site for sampling. In general, vestibular sites were preferred. These sites were also used for GCF and SP sampling, and the same sampling procedure was followed for all subjects. For the diseased sites, all samples were taken at the deepest sites (5 mm or more). This allowed us to explore the periodontal microbiota and the cytokine profile at 2 completely different levels (healthy and diseased). In practice, we performed the gingival fluid sampling first and the subgingival plaque sampling was performed at least 15 min later. Firstly, supragingival biofilm was removed, and the 3 healthy or diseased sites were isolated with cotton rolls to prevent contamination with saliva. GCF samples were carefully obtained from these 3 different healthy or diseased sites using 3 Periopaper strips^®^ (Oraflow, Smithtown, NY, USA) which were pooled in the same tube containing 120 µL of PBS Tween. Each paper strip was inserted into the sulcus or periodontal pocket and was left in place for 30 s before being removed. Strips that became contaminated by saliva or blood were excluded and a sample was taken again by changing the collection site. The GCF volume collected was measured using a calibrated Periotron 8000^®^ (Oraflow, Smithtown, NY, USA) and converted to real volume by reference to the standard curve [11]. For the subgingival plaque, samples were collected using paper points (protaper gold F3) which were inserted at 3 different diseased or healthy sites. Three paper points from these different sites were then pooled in the same dry sampling tube. A total of 144 sites (72 diseased sites and 72 healthy sites) were collected in the study. The fact that each patient acts as his or her own control limits the performance bias and inter-individual variability that can impact the study results. Thus, the variation in the composition of the microbiota and cytokines at healthy and diseased sites should follow the same dynamics in the same patient subjected to exogenous factors. All samples were immediately stored at the Biological Resources Center of Nantes Hospital at −80 °C.

### 2.5. Cytokine Quantification by Multiplexed Bead Immunoassay

The cytokine levels were determined using high-sensitivity human magnetic bead kits from Merck Millipore (St-Quentin-en-Yvelines, France), as well as a Luminex^®^ 100TM instrument and Bio-Plex Manager 6 software (Bio-Rad Laboratories, Hercules, CA, USA). The commercial 7-plex kits were used to analyze the sample levels of 7 cytokines (IL-1β, IL-2, IL-4, IL-6, TNF-α, IL-10, IL-17a) with the CIMNA platform of Nantes University Hospital according to the manufacturer’s instructions. These cytokines were measured in the samples and the results were calculated with Bio-Plex Manager Software (Bio-Rad Laboratories, Hercules, CA, USA).

### 2.6. Microbial Community Analysis

#### 2.6.1. Bacterial 16S rRNA Gene Amplification and Sequencing

The subgingival plaque samples collected using sterile paper points were kept frozen at −80 °C until they were processed. Samples were then immersed in 1.5 mL PBS inside a sterile microcentrifuge tube and vortexed to dislodge the bacteria. After removing the paper points with sterile cotton pliers, the samples were centrifuged at 20,000 rpm for 5 min to pellet the bacteria at room temperature, as previously described [7]. The DNA was then extracted with the QIAamp DNA Mini Kit (Qiagen Inc., Venlo, The Netherlands) following the manufacturer’s protocol. Amplicons spanning the variable region 4 of the bacterial 16S rRNA gene were generated and sequenced using Illumina Mi-seq platform at the University of Minnesota Genomic Center, Twin Cities, MN, USA [12].

#### 2.6.2. 16S Sequencing Data Analysis

The 16S rRNA gene sequencing data from the Illumina runs were then analyzed using the Quantitative Insights Into Microbial Ecology version 2 (QIIME2) software suite [13]. QIIME 2 computes error-corrected amplicon sequence variants (ASV) for Illumina read sequences. Primer-free sequences were imported into QIIME2 q2cli v2019.10, visually inspected with demux2, and processed via Deblur [14] to obtain the representative ASV sequences. Representative sequences and their abundances were extracted using the feature table [15]. A naive Bayes classifier [16] was fitted with 16S rRNA gene sequences extracted from Greengenes version 13_8 [17]. QIIME2 plugins were executed with standard parameters, and Deblur parameter “--p-trim-length” 240. Diversity analyses were also performed using the q2-diversity plugin, which computes alpha and beta diversity metrics, with a sampling depth of 4960. Alpha et beta diversity metrics were then analyzed and plotted using R version 4.0.3 [18], and using the ggplot2 [19]. Tests detecting the differences in beta diversity were performed using PERMANOVA, as implemented in R’s vegan package [20]. We also defined the core microbiome of the microbial communities in subgingival plaque samples of diseased and healthy sites [21].

### 2.7. Statistical Analysis

The data were analyzed using STATISTICA 12.0 (StatSoft, Inc, Tulsa, OK, USA). The descriptive statistics are presented as mean ± standard-deviation, minimum and maximum; and percentages (%) for categorical or ordinal parameters. We used Spearman correlation coefficient to analyze the link between a pro-inflammatory response and periodontal dysbiosis. Given the sample size, non-parametric tests were performed, using the Wilcoxon test for paired samples (healthy versus diseased sites) or the Mann–Whitney test for independent samples (tobacco Y/N).

For the microbiome analysis, differences between independent groups were respectively tested using a paired Mann–Whitney U test with False Discovery Rate correction. We also used the q2 picrust2 (Phylogenetic Investigation of Communities by Reconstruction of Unobserved States) plugin to predict the functional profiling of microbial communities using 16S rRNA marker gene sequences [22]. Heatmap correlations between genus relative abundances and the cytokines concentrations parameters were also calculated using the Spearman correlation coefficient. Statistical tests were considered significant for *p* < 0.05.

## 3. Results

### 3.1. Cytokine Profile Is Altered in Diseased Sites Compared to Healthy Sites

A total of 24 patients were recruited, consisting of 13 men (54%) and 11 women (46%). Twenty of them presented a periodontitis stage 3 (grade B or C) and 4 presented a periodontitis stage 4 (grade B or C). The stages of periodontitis were diagnosed on clinical charts and radiographic data according to the criteria of new classification. Demographic data, the mean of cigarettes smoked daily, and clinical parameters of the included patients are presented in Table 1. Six major cytokines, involved in periodontal inflammation, were measured and analyzed in GCF, i.e., IL-1β, IL-6, TNFα, IL-4, IL-10, IL-17a. IL-2 concentrations in GCF were likely under the detection level of the kit to be analyzed. As a conventional internal validation, the GCF amount collected in healthy sites were significantly lower than in diseased sites (Wilcoxon test, *p* < 0.05).

Sex, height, weight, and age did not significantly affect the cytokines profile. Likewise, IL-4, IL-6, IL-10 and IL-17A levels were not significantly modified by tobacco use, whether in healthy or diseased sites. However, tobacco use significantly increased the concentration of IL-1β in diseased sites (*p* < 0.05; Table 2), but not in healthy sites. Regarding TNF-α level, the concentration was significantly lower in healthy sites in smoking patients while the concentration in diseased sites did not significantly change (*p* < 0.05; Table 2). A comparison between healthy and diseased sites of the same oral cavity for IL-6 and TNF-α did not reveal any significant differences, whereas concentrations of IL-4, IL-1β, IL-17A, IL-10 were significantly different between diseased and healthy sites (Wilcoxon test *p* < 0.05; Table 3).

### 3.2. Microbiome Diversity Is Altered in Diseased Sites Compared to Healthy Sites

**Figure 1:**
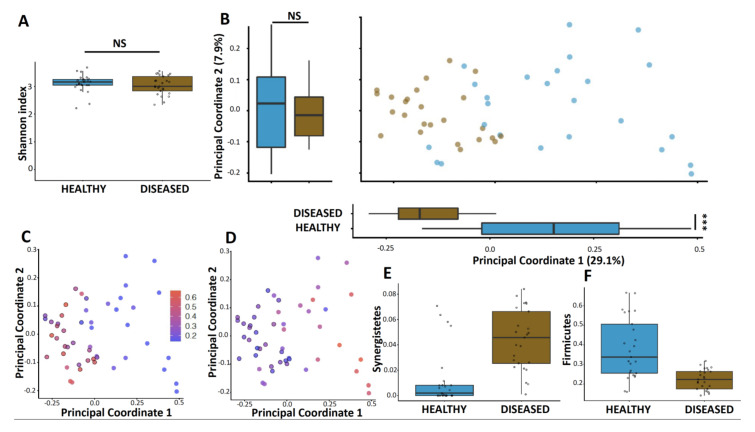
Microbiome diversity is altered in periodontitis sites compared to healthy sites. (**A**) Alpha diversity of periodontitis sites and healthy sites, depicted by the Shannon index, compared by the use of Mann-Whitney U test. (**B**) Principal-coordinate analysis of unweighted UniFrac distances for periodontitis sites and healthy sites. Box plots shown along each axis represent the median and interquartile range and indicate the distribution of samples along the given axis. Each point represents a single sample and is colored by site. PERMANOVA values, R2 values, and *p* values are shown. The same principal-coordinate analysis colored by the relative abundances of phyla, ***: *p* < 0.001 (**C**) Synergistetes and (**D**) Firmicutes. Relative abundances of phyla (**E**) Synergistetes and (**F**) Firmicutes within the periodontitis sites and the healthy sites, as assessed by Mann-Whitney tests with false-discovery-rate correction.

To understand how the oral microbiome changed according to periodontitis status, we analyzed bacterial DNA isolated from subgingival plaques of diseased and healthy sites of the same oral cavity in adults. Using non-phylogeny- and phylogeny-based alpha diversity metrics, we did not find a significant difference between diseased and healthy sites of sampling (Mann-Whitney U test, Shannon index, *p* > 0.05; observed amplicon sequence variants [ASV], *p* > 0.05; Figure 1A). However, the principal coordinate analysis of the unweighted UniFrac distances showed that the diseased and healthy sites of sampling clustered separately (permutational multivariate analysis of variance [PERMANOVA], R2 = 0.27, *p* < 0.001; Figure 1B). More specifically, the phylogenetic diversity in samples collected from diseased sites changed along principal coordinate one (Mann-Whitney U test, *p* < 0.001), whereas the healthy sites did not. The overall architecture was also significantly disrupted between diseased and healthy sites of sampling using weighted UniFrac distances (PERMANOVA, R2 = 0.68, *p* < 0.001). We also found that the clustering of subgingival plaque samples was caused, in part, by the increased relative abundances of Synergistetes in diseased versus healthy microbiomes (*p* < 0.001, Figure 1C,E) as well as an increased abundance of Firmicutes in healthy versus diseased samples (*p* < 0.001, Figure 1D,F) (Mann-Whitney U test, false-discovery rate [FDR] adjusted). Moreover, a compositional biplot, that simultaneously displays the sample clustering and the important taxa, identified that the healthy sites were mainly driven by Firmicutes and TM7 (Figure S1), whereas diseased sites were mainly driven by Synergistetes and Bacteroidetes (Figure S2).

### 3.3. Microbiome of Diseased Sites Was Enriched in Synergistetes and Bacteroidetes and Depleted in Firmicutes and Proteobacteria

**Figure 2:**
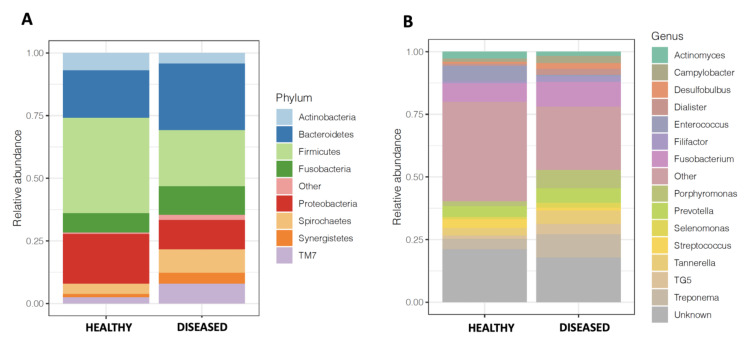
Taxonomic profile of the oral microbiomes of the samples collected in periodontitis sites and healthy sites. (**A**) Mean relative taxa abundance plots for individuals from the samples collected in periodontitis sites and healthy sites, summarized at the phylum level. (**B**) Mean relative taxa abundance plots for individuals from the samples collected in periodontitis sites and healthy sites, summarized at the genus level.

We then explored the taxonomic differences between diseased and healthy sites of sampling. Overall, at phylum level, diseased sites were dominated by Bacteroidetes (relative abundance, mean ± standard deviation, 0.19 ± 0.09 in healthy sites versus 0.27 ± 0.06; Mann-Whitney U test, *p* value FDR adjusted < 0.001), whereas healthy sites were dominated by Firmicutes (relative abundance, mean ± standard deviation, 0.38 ± 0.15 in healthy sites versus 0.22 ± 0.05; Mann-Whitney U test, *p* value FDR adjusted < 0.001) (Figure 2A and Table S1A). Moreover, we found that diseased sites were enriched in Synergistetes, TM7, SR1, Spirochaetes, Bacteroidetes and Fusobacteria, and depleted in Firmicutes, Proteobacteria, Tenericutes and Actinobacteria compared to healthy sites (Mann-Whitney U test, *p* value FDR adjusted < 0.10, Table S1A). At the genus level, we found that diseased sites were enriched in *Schwartzia*, *Porphyromonas*, *Mogibacterium*, *Prevotella*, *Dialister*, *Selenomonas*, *Eubacterium* and *Peptostreptococcus*, whereas healthy sites were enriched in *Enterococcus*, *Pseudomonas*, unclassified Enterobacteriaceae, and *Propionibacterium* (Figure 2B and Table S1B). We also defined, at ASV level, the core microbiome in diseased and healthy sites. In diseased sites, the core microbiome was composed of 55 ASV, including *Porphyromonas endodontalis*, *Treponema socranskii* and *Veillonella dispar*, whereas the core microbiome in healthy sites was composed of 25 ASV, including *Novosphingobium capsulatum*, and several members of genus *Actinomyces* (Table S1C).

### 3.4. Prediction of Functional Profiles Using 16S rRNA Data in Diseased Sites Compared to Healthy Sites

To generate a functional profile of subgingival plaques in diseased and healthy sites, PICRUSt was used to predict metagenomic functions by modelling genes from 16S rRNA data. To gain insight into the metabolic contribution of bacteria to the subgingival plaques’ ecosystem, the prediction tool PICRUSt was used to determine the functional characteristics of the bacterial communities in the subgingival plaque’s healthy and diseased sites. Metagenomic reads were then assigned to the MetaCyc pathways [23] and Enzyme Commission (EC) [24]. Using alpha diversity metrics, we did not find a significant difference between functional profiles of diseased and healthy sites of sampling (Mann-Whitney U test, Shannon index, *p* = 0.40, Figure S3A; observed Pathways, *p* = 0.16, Figure S3B). The overall functional architecture was significantly disrupted between diseased and healthy sites of sampling using Bray Curtis distances (PERMANOVA, R2 = 0.20, *p* value < 0.001, Figure S4).

We found that diseased sites were enriched with CODH-PWY (reductive acetyl coenzyme A pathway I), PWY-7374 (1,4-dihydroxy-6-naphthoate biosynthesis I), WY-6263 (superpathway of menaquinol-8 biosynthesis II), PWY-7371 (1,4-dihydroxy-6-naphthoate biosynthesis II), PWY-7373 (superpathway of demethylmenaquinol-6 biosynthesis II), PWY-7528 (L-methionine salvage cycle I), THISYN-PWY (superpathway of thiamine diphosphate biosynthesis I), P163-PWY (L-lysine fermentation to acetate and butanoate), PWY-6545 (pyrimidine deoxyribonucleotides de novo biosynthesis III), P162-PWY (L-glutamate degradation V) and PWY-6892 (thiazole biosynthesis I) pathways, compared to healthy sites (Mann-Whitney U test, *p* value FDR adjusted < 0.05, Table S1D). We then defined, at the MetaCyc pathway level, the core microbiome in diseased and healthy sites. In diseased sites, the core microbiome was composed of 221 pathways, among them 7 were found only in diseased sites, namely, the superpathway of Clostridium acetobutylicum acidogenic fermentation, superpathway of menaquinol-8 biosynthesis II, L-lysine fermentation to acetate and butanoate, fucose degradation, reductive acetyl coenzyme A pathway, pyruvate fermentation to butanoate and succinate fermentation to butanoate. In healthy sites, the core microbiome was composed of 236 pathways, among them 22 were found only in healthy sites, including enterobactin biosynthesis, fatty acid & beta;-oxidation I, glucose and glucose-1-phosphate degradation, glyoxylate cycle, superpathway of (Kdo)2-lipid A biosynthesis, lactose and galactose degradation I and L-leucine degradation I (*p* value FDR adjusted < 0.05, Table S1E). We also found significant differences in the observed proportions of enzyme types between diseased and healthy sites, including enrichment in glutamate and sulfur metabolism in diseased sites (*p* value FDR adjusted < 0.05, Table S1E).

### 3.5. Host-Microbiome Interaction Is Altered in Diseased Sites Compared to Healthy Sites

To detect the specific interactions, correlations were performed between cytokines profile and the oral microbiome. We did not find any significant correlation between IL1-Beta concentration and the Shannon index (r = 0.28, *p* = 0.18), and between alpha diversity measures (expressed using the Shannon and Simpson indexes) and pro-inflammatory cytokines (IL-1β, IL-2, IL-6, TNFα, IL-4, IL-10, IL-17). However, we noticed a trend between TNFα and the Shannon index (r = −0.35; *p* = 0.09).

**Figure 3:**
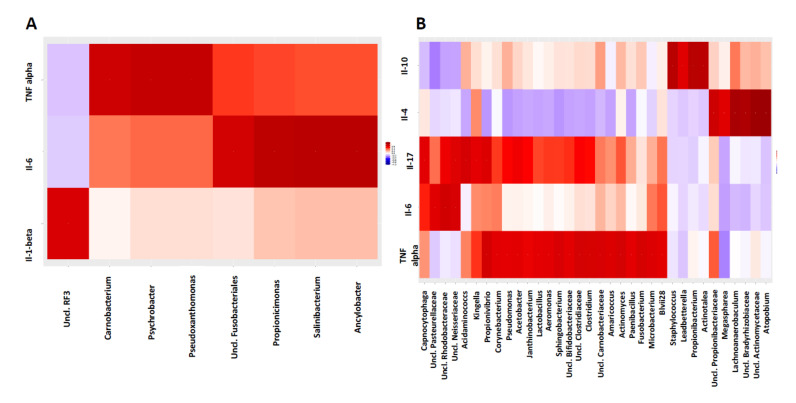
Correlation heatmaps for cytokines and oral microbiomes collapsed at genus level in the samples collected in (**A**) healthy sites and in (**B**) periodontitis sites. Increasing values are translated into colors from blue (negative correlation) to red (positive correlation).

In the healthy sites, we observed significant correlations between IL-6 and *Fusobacterium, Salinibacterium, Enterococcus* and between *TNF alpha* and *Streptococcus, Actinomyces* and *Propionicimonas.* In diseased sites, we observed significant correlations between Il-4 and *Prevotella, Atopobium rimae, Propionibacterium, Lachnoanaerobaculum orale,* Il-10 and *Propionibacterium* and *Staphylococcus,* between *TNF alpha* and *Actinomyces* and between *Clostridium* and *Fusobacterium* (*p* value FDR adjusted < 0.05, Figure 3 and Table S2A). We also found that IL-17A was significantly correlated with BENZCOA-PWY (anaerobic aromatic compound degradation), DENITRIFICATION-PWY (nitrate reduction I), and PWY-7402 (benzoate fermentation), and IL-6 was significantly correlated with PWY-5499 (vitamin B6 degradation) and PWY-7402 (benzoate fermentation) (*p* value FDR adjusted < 0.05, Table S2B). We also found significant correlations of EC 1.14.19.4 (delta(8)-fatty-acid desaturase), EC 3.4.23.49 (Omptin) and EC 4.1.2.55 aldolase and IL-6 (*p* value FDR adjusted < 0.10, Table S2C–F).

## 4. Discussion

In this work, we aimed to assess the microbiota composition and cytokine profile changes in patients with stage III/IV, grade B/C periodontitis, by comparing healthy and diseases sites in the same oral cavity. Periodontitis is characterized by a destruction of the attachment apparatus as a consequence of an interaction between a dysbiotic subgingival microbial community and the immunoinflammatory response of the host. It is well established that patients with periodontitis have both healthy and affected sites [25], and our study was the first to assess both cytokine and oral microbiome profiles simultaneously in the same oral cavity.

We then analyzed cytokine and oral microbiome profiles in healthy and diseased sites (intra-individual) in patients with acute periodontitis stage III or IV and grade B/C. Cytokines levels were expressed in pg/mL per site and were correlated with smoking. We chose to include smokers in this study because the proportion of smokers in the general population of patients with periodontal disease is significant. Usually subgingival samples are collected using curettes, but we used paper tips because the use of curettes is more traumatic than paper points and causes bleeding more easily at inflammatory sites.

If we were unable to highlight correlations between the cytokines expression and dysbiosis indexes, our results showed that Interleukin-1b, Interleukin-4, Interleukin-10, and Interleukin-17A were significantly overexpressed in diseased sites, whereas Interleukin-6 and TNF-alpha did not differ significantly between healthy and diseased sites. The choice of the cytokines panel is justified because of their role in the initiation and progression of periodontal disease, including the role of pro and anti-inflammatory cytokines. Previous findings reported increased IL-6 expression in chronic periodontitis [26]. Another study reported an IL-6 gene polymorphism, which is considered as a susceptibility factor for chronic periodontitis in a Chinese population [27]. The same was found for TNF-alpha, as Ying Li et al. [28] reported that genetic polymorphism (rs 361525, 1800629 and 1799964) can influence the predisposition to the development of periodontitis, and has only been associated with aggressive periodontitis by Cheng Ding et al. [29].

The concentration of other cytokines (IL-4, 10, 17-A) was less variable in both healthy and diseased sites, and the results for IL-1βt are entirely consistent with the literature due to its proinflammatory nature. However, it is established that the composition of the microbial biofilm has an influence on the composition and concentration of the various cytokines in the gingival fluid. Furthermore, Van Dyke suggested that the immunoinflammatory response drives the composition of the bacterial biofilm and the emergence of pathogens [30].

We were not able to correlate cytokine concentrations to alpha-diversity measures or other microbiome structural indexes. However, as reported by Zhou et al. [9] the beta-diversity comparisons, evaluated using weighted and unweighted UniFrac distances, revealed significant differences in the level of oral microbiota present between healthy and periodontitis sites in the same oral cavity. These results agree with those described by Kistler et al. [31], who compared healthy subjects and subjects with periodontitis, and by Camelo-Castillo et al. [32], who observed a significantly different microbial structure between health, non-smoking-associated periodontitis and smoking-associated periodontitis. We also found that clustering between healthy and diseased sites was mainly caused by the relative abundance of the phyla Synergistetes and Firmicutes. We confirmed findings from Zhou et al. that showed that oral microbiota from periodontitis sites had a higher relative abundance of Spirochetes and Synergistetes and that oral microbiota from healthy sites had a higher relative abundance of Actinobacteria. Moreover, we found that periodontitis sites were enriched in Bacteroidetes and depleted in Firmicutes. Importantly, we confirmed that the following 3 genera; *Desulfobulbus*, *Filifactor* and *TM7* were significantly associated with the disease. Patini et al. [33] considered these taxa as potential biomarkers of periodontitis as they were found in at least 5 of the studies included in their review. Importantly, Cross et al. [34] recently demonstrated that *Desulfobulbus oralis* can trigger a proinflammatory response in oral epithelial cells, suggesting its direct role in the development of periodontal disease. *Filifactor alocis*, a Gram-positive anaerobic rod, is frequently identified in periodontitis, and Anuri et al. [35] demonstrated that this strain had virulence properties that enhance its ability to survive and persist in the periodontal pocket, thus playing an important role in an infection-induced periodontal disease. *TM7* was previously found associated with periodontitis [36] and with inflammatory bowel disease [37], confirming the potential role of this subgroup of gram-positive uncultivated bacteria in the inflammatory pathogenesis of periodontitis [38]. We also found that *Fusobacterium* may play a pivotal role in pathogenesis of periodontitis by favoring bacteria attachment in the periodontal biofilm, as previously reported by Camelo-Castillo et al. [32]. In our study we also reported the well-described taxa associated with periodontitis including *Porphyromonas*, *Tannerella* and *Treponema* [39], by comparing healthy and diseased sites in the same oral cavity.

Our study also demonstrates that the altered oral microbiota in diseased sites affected the cytokines levels, demonstrating a significant positive correlation between pro-inflammatory TNF-α and taxa known to be associated with pathogenesis of periodontitis, such as *Actinomyces*, and *Fusobacterium*. We also found significant positive correlations between anti-inflammatory cytokines, especially Il-4 and Il-10 and bacteria known to be associated with oral health conditions, such as *Atopobium rimae*, *Propionibacterium*, and *Lachnoanaerobaculum orale*. Zhou et al. [9] previously reported that *Fusobacterium nucleatum* and *Actinomyces* affect cytokine levels, revealing that both the altered oral microbiota and altered cytokine profile may be pivotal for the pathogenesis of periodontitis. We also found that pro-inflammatory IL-17A was significantly correlated with nitrate reduction I, a pathway known to be associated with intestinal inflammation [40]. We also found significant correlations between enzymes known to be associated with inflammation and Il-6, such as EC 1.14.19.4 (delta(8)-fatty-acid desaturase) [41]. We found that tobacco use only significantly increased the concentration of IL-1β in diseased sites. BinShabaib et al. [42] reported in their study, of which the aim was to compare the clinical periodontal status and gingival crevicular fluid (GCF) cytokine profile among cigarette-smokers (Group-1), electronic-cigarette users (Group-2) and never-smokers (Group-3), that periodontal status was poorer and the concentrations of IL-1β, IL-6, IFN-γ, TNF-α and MMP-8 were significantly higher in the GCF samples of individuals in Group-1 (*p* < 0.05) than in groups 2 and 3 [42]. We did not evaluate the effect of tobacco on subgingival microbiota, although other authors such as Mager et al. [43] have already shown in their investigation that periodontal pathogens were found at higher levels on the soft tissues of periodontitis subjects than in periodontally healthy subjects, and at higher levels in smokers than nonsmokers [43]. However, these were not found to be statistically significant.

However, our study has several limitations. First, we included patients with periodontitis stage III or VI and grade B/C, but a comparison between the two stages cannot be made due to our limited sample size. Second, we selected the panel of cytokine that were measured. Future works, including a meta-analysis of periodontitis patients from other geographical areas, may be of interest to develop a better understanding of the microbiome composition and its influence on the cytokine profile [9].

## 5. Conclusions

Here, we observed concomitant changes in subgingival plaque microbiota and the cytokine profile, suggesting that this combined alteration could be a factor in the pathobiology of periodontitis. Further studies should assess whether the manipulation of the dysbiotic oral microbiota is able to provide better protection against periodontal disease.

## Figures and Tables

**Table 1:** Demographic and clinical data of the 24 included participants.

Demographic and Clinical Parameters	Patients Characteristics (N = 24)
Age Mean ± SD (Min-Max)	49.70 ± 13.26 (28–75)
BMI Mean ± SD (Min-Max)	25.77 ± 4.17 (19.4–36.5)
Sex % Men (n/N)	54.2% (13/24)
Teeth Number Mean ± SD (Min-Max)	25.75 ± 3.29 (19–31)
Bacterial Plaque Index (FMPS) Mean ± SD (Min-Max)	47.58 ± 18.41 (11–78)
Bleeding Index (FMBS) Mean ± SD (Min-Max)	23.04 ± 10.57 (1–42)
Pocket Depth in mm Mean ± SD (Min-Max)	3.67 ± 0.60 (2.6–5.3)
Clinical Attachment Level in mm Mean ± SD (Min-Max)	4.57 ± 1.23 (3.2–8)
Tobacco Consumption Mean ± SD (Min-Max)	8.7 ± 8.87 (0.29-30)
Periodontal Disease Severity (According PAPAPANOU Classification) % (n/N)	Stage III grade B—71% (17/24)
	Stage III grade C—12.5% (3/24)
	Stage IV grade B—8.3% (2/24)
	Stage IV grade C—8.3% (2/24)

SD = Standard Deviation.

**Table 2:** Comparison between GCF cytokines and the smoking status.

Cytokines Median (Min-Max)	Site/Ratio	Tobacco (N = 11)	No Tobacco (N = 13)	*p*-Value
IL-10	Healthy	4.76 (1.7–5.9)	4.95 (3.2–6.3)	>0.05
	Diseased	5.93 (4.8–15.1)	6.71 (4.4–9.1)	>0.05
	D/H ratio	1.61 (1–4.7)	1.29 (0.9–2.1)	>0.05
IL-17A	Healthy	1.02 (0.2–2)	1.12 (0.4–2.3)	>0.05
	Diseased	1.31 (0.8–3.2)	1.71 (1–4.8)	>0.05
	D/H ratio	1.41 (0.7–5.5)	1.90 (1–11.4)	>0.05
IL-1β	Healthy	193.4 (5.1–673.9)	62.09 (3.3–449.5)	<0.05
	Diseased	679.94 (322.7–911.8)	382.75 (122.9–836.9)	<0.05
	D/H ratio	3.52 (0.7–179.1)	6.28 (1.2–79.9)	>0.05
IL-4	Healthy	2.74 (2.4–3.8)	2.40 (2.4–3.8)	>0.05
	Diseased	3.09 (3–4.5)	3.09 (2.4–9.5)	>0.05
	D/H ratio	1.28 (0.8–1.5)	1.29 (1–3.9)	>0.05
IL-6	Healthy	1.76 (0.7–6.4)	3.52 (0.6–40.1)	>0.05
	Diseased	2.4 (0.6–15.6)	2 (0.9–36.6)	>0.05
	D/H ratio	0.87 (0.2–21.4)	0.65 (0.3–3.9)	>0.05
TNF-Alpha	Healthy	2.91 (1.3–10.4)	4.90 (2.2–25.7)	<0.05
	Diseased	1.46 (2.1–15.7)	6.31 (1.3–27.6)	>0.05
	D/H ratio	1.80 (0.3–6.7)	0.64 (0.2–7.1)	>0.05

GCF: Gingival crevicular fluid GCF.

**Table 3:** Cytokines concentration comparison between healthy and diseased sites.

Cytokines Median (Min-Max)	Healthy Sites (N = 24)	Diseased Sites (N = 24)	*p*-Value
IL-10	4.86 (1.7–6.3)	6.71 (4.4–15.1)	<0.05
IL-17a	1.02 (0.2–2.3)	1.53 (0.8–4.8)	<0.05
IL-1β	68.59 (3.3–673,9)	500.26 (122.9–911.8)	<0.05
IL-4	2.4 (2.4–3.8)	3.09 (2.4–9.5)	<0.05
IL-6	2.12 (0.6–40.1)	2.20 (0.6–36.6)	>0.05
TNFα	4.54 (1.3–25.7)	4.85 (1.3–27.5)	>0.05
Concentration of Total Cytokines	84.27 (14.7–685.4)	529.24 (174.8–933.6)	<0.05

## Data Availability

The data set generated and analyzed for the current study is available in the NCBI repository under the primary accession number BioProject ID PRJNA744078 (http://www.ncbi.nlm.nih.gov/bioproject/744078 accessed on 15 November 2021).

## Supplementary Materials

The following are available online at https://www.mdpi.com/article/10.3390/microorganisms9112364/s1. Table S1: (A) Relative abundance of the most significant phyla in the samples collected in periodontitis sites and healthy sites as assessed by Mann-Whitney U tests with false-discovery-rate correction. (B) Relative abundance of the most significant genera in the samples collected in periodontitis sites and healthy sites as assessed by Mann-Whitney U tests with false-discovery-rate correction. (C) Relative abundance of the most significant ASV in the samples collected in periodontitis sites and healthy sites as assessed by Mann-Whitney U tests with false-discovery-rate correction. (D) Relative abundance of the most significant KEGG metabolic pathways in the samples collected in periodontitis sites and healthy sites as assessed by Mann-Whitney U tests with false-discovery-rate correction. (E) Relative abundance of the most significant MetaCyc metabolic pathways in the samples collected in periodontitis sites and healthy sites as assessed by Mann-Whitney U tests with false-discovery-rate correction. Table S2: (A) Most significant correlations between cytokines and oral microbiome collapsed at genus level in the samples collected in healthy sites as assessed by Pearson correlation with false-discovery-rate correction. (B) Most significant correlations between cytokines and oral microbiome collapsed at genus level in the samples collected in periodontitis sites as assessed by Pearson correlation with false-discovery-rate correction. (C) Most significant correlations between cytokines and predicted metagenomic functions (KEGG pathways) in the samples collected in healthy sites as assessed by Pearson correlation with false-discovery-rate correction. (D) Most significant correlations between cytokines and predicted metagenomic functions (KEGG pathways) in the samples collected in periodontitis sites as assessed by Pearson correlation with false-discovery-rate correction. (E) Most significant correlations between cytokines and predicted metagenomic functions (Enzyme Commission) in the samples collected in healthy sites as assessed by Pearson correlation with false-discovery-rate correction. (F) Most significant correlations between cytokines and predicted metagenomic functions (Enzyme Commission) in the samples collected in periodontitis sites as assessed by Pearson correlation with false-discovery-rate correction. Figure S1: Compositional biplot, that simultaneously displays the sample clustering, with each point representing a single sample of the healthy sites, and the important taxa identified at phylum level This plot showed that healthy sites samples were mainly driven by Firmicutes and TM7. Figure S2: Compositional biplot, that simultaneously displays the sample clustering, with each point representing a single sample of the healthy sites, and the important taxa identified at phylum level. This plot showed that diseased samples were mainly driven by Synergistetes and Bacteroidetes. Figure S3: Alpha diversities of the predict metagenomic functions of the samples collected in periodontitis sites and healthy sites using (A) the total numbers of observed pathways and (B) the Shannon index. Whiskers in the boxplot represent the range of minimum and maximum alpha diversity values within a population, excluding outliers. No tests yielded a significant difference. Figure S4: Functional diversity of the oral microbiomes of the predict metagenomic functions of the samples collected in periodontitis sites and healthy sites. Principal Coordinates Analysis of Bray–Curtis distances generated from pathways table. Proportion of variance explained by each principal coordinate axis is denoted in the corresponding axis label. The PCoA shows clear separation between faecal samples collected before chemotherapy and after chemotherapy.
