# Supplementary material for: Subgingival Microbiota and Cytokines Profile Changes in Patients with Periodontitis: A Pilot Study Comparing Healthy and Diseased Sites in the Same Oral Cavities
Source: Microorganisms. 2021 Nov 16;9(11):2364. doi: 10.3390/microorganisms9112364 (PMC8618247; doi:10.3390/microorganisms9112364)
Supplement: Supplementary file 1 [file microorganisms-09-02364-s001.zip › microorganisms-1427995-Supplementary.pdf]

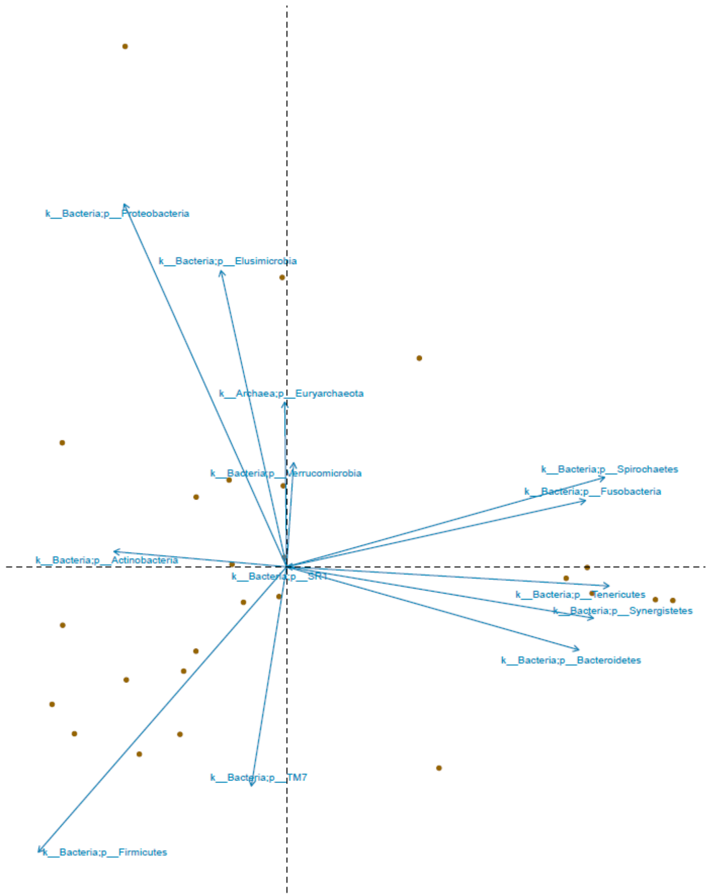

Figure S1 - Compositional biplot, that simultaneously displays the sample clustering, with each point representing a single sample of the healthy sites, and the important taxa identified at phylum level. This plot showed that healthy sites samples were mainly driven by Firmicutes and TM7.

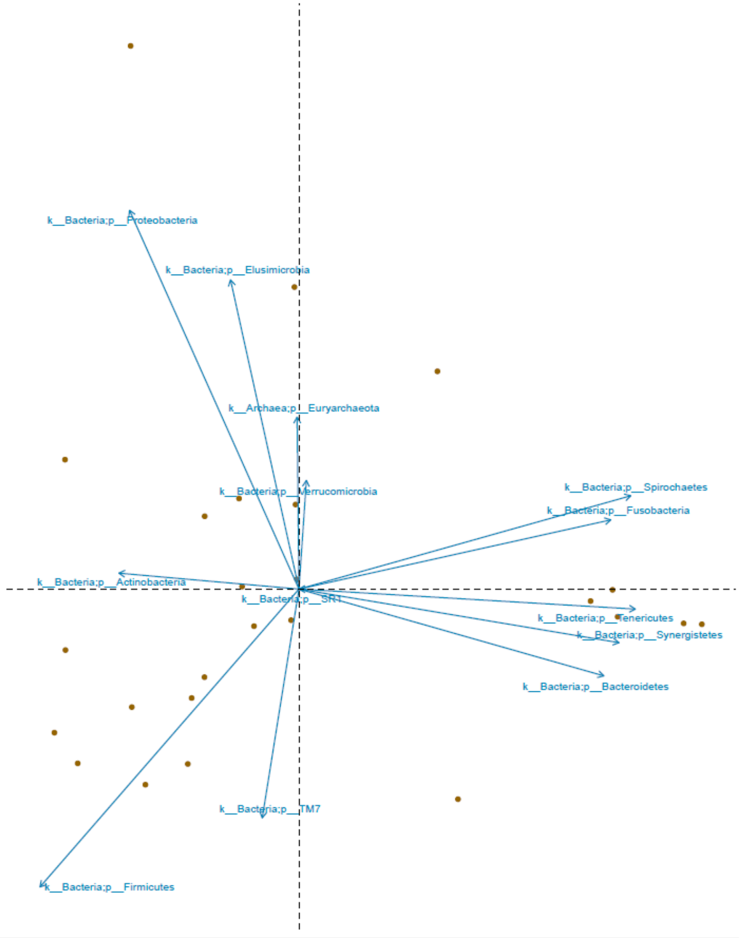

Figure S2 - Compositional biplot, that simultaneously displays the sample clustering, with each point representing a single sample of the healthy sites, and the important taxa identified at phylum level. This plot showed that diseased samples were mainly driven by Synergistetes and Bacteroidetes.

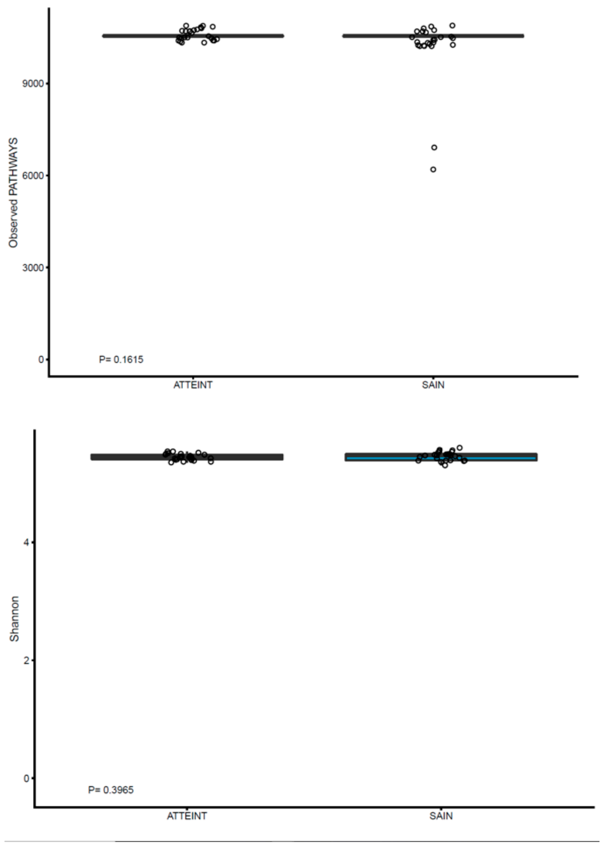

Figure S3 - Alpha diversities of the predict metagenomic functions of the samples collected in periodontitis sites and healthy sites using (**A**) the total numbers of observed pathways and (**B**) the shannon index. Whiskers in the boxplot represent the range of minimum and maximum alpha diversityvalues within a population, excluding outliers. No tests yielded a significant difference.

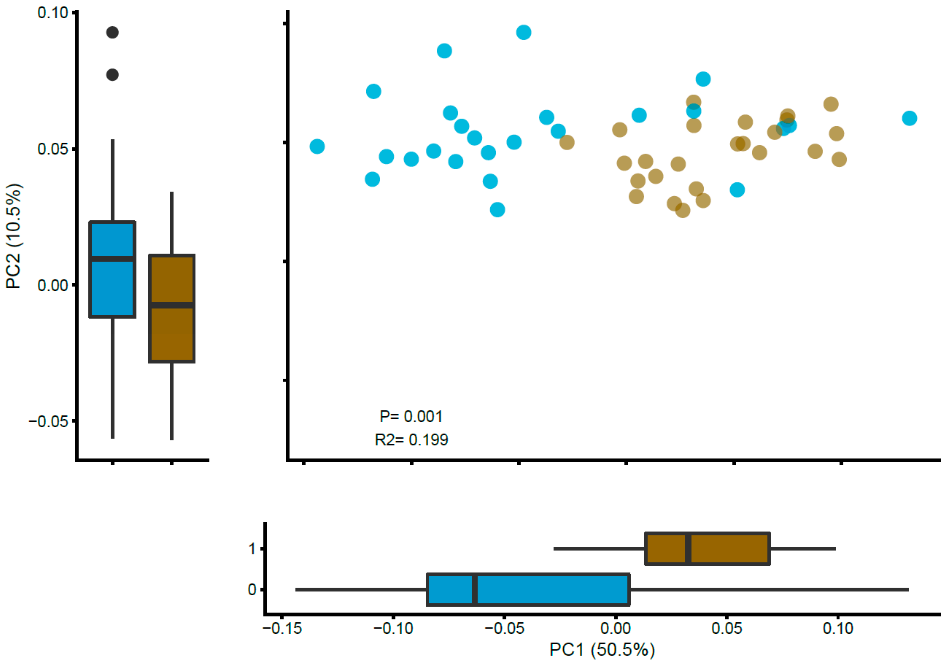

Figure S4 - Functional diversity of the oral microbiomes of the predict metagenomic functions of the samples collected in periodontitis sites and healthy sites. Principal Coordinates Analysis of Bray–Curtis distances generated from pathways table. Proportion of variance explained by each principal coordinate axis is denoted in the corresponding axis label. The PCoA shows clear separation between faecal samples collected before chemotherapy and after chemotherapy.

Feature	P value	FDR corrected P value	mean relative abundance healthy sites	mean relative abudance diseased sites
k__Bacteria;p__Synergistetes	0.0000127963181505418	0.000204741090408669	0.0130342887773651	0.0436740765836695
k__Bacteria;p__Firmicutes	0.0000887786327270478	0.000473486041210921	0.381067316933096	0.223589012028169
k__Bacteria;p__TM7	0.000478348082313356	0.0017440863141694	0.0245489811479369	0.078776742131996
k__Bacteria;p__SR1	0.000545026973177937	0.0017440863141694	0	0.00215848238376248
k__Bacteria;p__Spirochaetes	0.000780787451612888	0.0020820998709677	0.0415370521086175	0.0937275137812838
k__Bacteria;p__Bacteroidetes	0.00280773814974955	0.00641768719942754	0.189027028601388	0.26581083203824
k__Bacteria;p__Proteobacteria	0.0198939507675555	0.0397879015351109	0.198693091678517	0.116957110402858
k__Bacteria;p__Tenericutes	0.0249433697703493	0.044343768480621	0.00237656679120939	0.00412197754699422
k__Bacteria;p__Fusobacteria	0.0378658098717794	0.060585295794847	0.0765980599127453	0.114318718130449
k__Bacteria;p__Actinobacteria	0.0572392612793856	0.0832571073154699	0.0699247819222139	0.0427055644726608

Table S1A. Relative abundance of the most significant phyla in the samples collected in periodontitis sites and healthy sites as assessed by Mann-Whitney U tests with false-discovery-rate correction.

Feature	P value	FDR corrected P value	mean relative abundance healthy sites	mean relative abudance diseased sites
k__Bacteria;p__Firmicutes;c__Clostridia;o__Clostridiales;f__Veillonellaceae;g__Schwartzia	0.0000100791304482579	0.00156341723298603	0.00185557988957733	0.00750991065434733
k__Bacteria;p__Synergistetes;c__Synergistia;o__Synergistales;f__Dethiosulfovibrionaceae;g__TG5	0.0000198867513278845	0.00156341723298603	0.0130342887773651	0.0412271032692709
k__Bacteria;p__TM7;c__TM7-3;o__;f__;g__	0.0000203041199089094	0.00156341723298603	0.010056874230823	0.0661263239468699

k__Bacteria;p__Firmicutes;c__Bacilli;o__Lactobacillales;f__Enterococcaceae;g__Enterococcus	0.0000423272928012977	0.00197192320788367	0.0611968347072659	0.00676404580854227
k__Bacteria;p__Proteobacteria;c__Gammaproteobacteria;o__Pseudomonadales;f__Pseudomonadaceae;g__Pseudomonas	0.0000539704245459434	0.00197192320788367	0.0218921164835223	0.00173840752749208
k__Bacteria;p__Proteobacteria;c__Gammaproteobacteria;o__Enterobacteriales;f__Enterobacteriaceae;__	0.0000715050908165901	0.00197192320788367	0.0176239620787316	0.00200425773198034
k__Bacteria;p__TM7;c__TM7-3;o__I025;f__Rs-045;g__	0.0000737874573352613	0.00197192320788367	0.00149695700404925	0.00625780016465631
k__Bacteria;p__Bacteroidetes;c__Bacteroidia;o__Bacteroidales;f__Porphyromonadaceae;g__Porphyromonas	0.0000744767000457875	0.00197192320788367	0.0193267612260351	0.0728691569606997
k__Bacteria;p__Firmicutes;c__Clostridia;o__Clostridiales;f__Peptostreptococcaceae;g__Filifactor	0.0000768281769305324	0.00197192320788367	0.0084088476681549	0.0208988469218348
k__Bacteria;p__Actinobacteria;c__Coriobacteriia;o__Coriobacteriales;f__Coriobacteriaceae;__	0.0000960631906447407	0.00205408678164307	0.00093160350420155	0.00329455215196643
k__Bacteria;p__Chloroflexi;c__Anaerolineae;o__Anaerolineales;f__Anaerolinaceae;g__SHD-231	0.000105386473184199	0.00205408678164307	0.0012735961597049	0.00810252460778169
k__Bacteria;p__Firmicutes;c__Clostridia;o__Clostridiales;f__[Mogibacteriaceae];g__Mogibacterium	0.000106705806838601	0.00205408678164307	0.00500955067723661	0.00950382500509464
k__Bacteria;p__Bacteroidetes;c__Bacteroidia;o__Bacteroidales;f__Rikenellaceae;g__Blvii28	0.000117602176056931	0.00207150644368834	0.00175928203576694	0.00765227423061183
k__Bacteria;p__Bacteroidetes;c__Bacteroidia;o__Bacteroidales;f__[Paraprevotellaceae];g__[Prevotella]	0.000125545845072021	0.00207150644368834	0.00209491780584918	0.0183025040253202
k__Bacteria;p__Actinobacteria;c__Actinobacteria;o__Actinomycetales;f__Propionibacteriaceae;g__Propionibacterium	0.000154909027828394	0.00238559902855727	0.00163292109898592	0.000359036613051911

k__Bacteria;p__Proteobacteria;c__Deltaproteobacteria;o__Desulfobacterales;f__Desulfobulbaceae;g__Desulfobulbus	0.000199863763431739	0.00288553308454574	0.00719430458265305	0.0235958213627634
k__Bacteria;p__Firmicutes;c__Clostridia;o__Clostridiales;f__Veillonellaceae;g__Dialister	0.000234955988619876	0.0031926372571289	0.00883658172068227	0.0234966617440765
k__Bacteria;p__Firmicutes;c__Clostridia;o__Clostridiales;f__Eubacteriaceae;g__Pseudoramibacter_Eubacterium	0.000281801603747826	0.00361645391476377	0.000280076484804709	0.00086499582039246
k__Bacteria;p__Firmicutes;c__Erysipelotrichi;o__Erysipelotrichales;f__Erysipelotrichaceae;g__Bulleidia	0.000305434893943592	0.00371344528952473	0.000451463691167561	0.00214335579294333
k__Bacteria;p__SR1;c__;o__;f__;g__	0.000545026973177937	0.0060804388400556	0	0.00215848238376248
k__Bacteria;p__Firmicutes;c__Bacilli;o__Lactobacillales;__;__	0.000552767167277782	0.0060804388400556	0.0544241252642022	0.00754594917662256
k__Bacteria;p__Tenericutes;c__RF3;o__ML615J-28;f__;g__	0.000651453681605986	0.00663728803059029	0.00009247552391704	0.00051754399701 7256
k__Bacteria;p__Firmicutes;c__Bacilli;o__Bacillales;f__[Exiguobacteraceae];g__	0.000660855518197302	0.00663728803059029	0.0199636232701272	0.00169856139395153
k__Bacteria;p__Firmicutes;c__Clostridia;o__Clostridiales;f__Veillonellaceae;g__Selenomonas	0.000709542314277409	0.00682934477492006	0.00895332387815447	0.0197580145490676
k__Bacteria;p__Spirochaetes;c__Spirochaetes;o__Spirochaetales;f__Spirochaetaceae;g__Treponema	0.000780787451612888	0.00721447605290309	0.0414886629579744	0.0934090274101671
k__Bacteria;p__Firmicutes;c__Clostridia;o__Clostridiales;f__Lachnospiraceae;g__Moryella	0.000864092326060033	0.00754700663591315	0.000282816360263572	0.00448395048373287
k__Bacteria;p__Firmicutes;c__Clostridia;o__Clostridiales;f__Ruminococcaceae;g__Ethanoligenens	0.000897987508512424	0.00754700663591315	0.0172419272400075	0.00241921949584356

k__Bacteria;p__Firmicutes;c__Clostridia;o__Clostridiales;f__[Acidaminobacteraceae];g__	0.000914788683140988	0.00754700663591315	0.00336120593726106	0.00801066822739141
k__Bacteria;p__Firmicutes;c__Clostridia;o__Clostridiales;f__[Mogibacteriaceae];g__	0.0010385521365436	0.00827260494970939	0.0114692732410886	0.0170527643927847
k__Bacteria;p__Proteobacteria;c__Epsilonproteobacteria;o__Campylobacterales;f__Campylobacteraceae;g__Campylobacter	0.00110464768416196	0.00850578716804706	0.0131674572575732	0.0282356117550576
k__Bacteria;p__Actinobacteria;c__Coriobacteriia;o__Coriobacteriales;f__Coriobacteriaceae;g__Slackia	0.00119215586622122	0.00888348403539036	0.000262694602775136	0.0011429658822502
k__Bacteria;p__TM7;c__TM7-3;o__CW040;f__F16;g__	0.00139400804216599	0.0100629955543857	0.000134691877798137	0.00188922550136516
k__Bacteria;p__Firmicutes;c__Clostridia;o__Clostridiales;f__;g__	0.00158075954125632	0.0110653167887943	0.00261309822701033	0.00699003160414388
k__Bacteria;p__Firmicutes;c__Clostridia;o__Clostridiales;f__Lachnospiraceae;g__Catonella	0.00175027847544265	0.0118915978772721	0.000706099407784039	0.00144059241831715
k__Bacteria;p__Firmicutes;c__Clostridia;o__Clostridiales;f__Peptostreptococcaceae;g__Peptostreptococcus	0.00191669368786674	0.0126501783399205	0.000402344861323961	0.00146229645367025
k__Bacteria;p__Actinobacteria;c__Coriobacteriia;o__Coriobacteriales;f__Coriobacteriaceae;g__Olsenella	0.00208035109962183	0.0133489195559067	0.000549399540526134	0.00140050420968871
k__Bacteria;p__Firmicutes;c__Clostridia;o__Clostridiales;f__Lachnospiraceae;g__Clostridium	0.00236766434200015	0.0147819044054604	0.00261768053296383	0.0000581791884145145
k__Bacteria;p__Firmicutes;c__Erysipelotrichi;o__Erysipelotrichales;f__Erysipelotrichaceae;g__Sharpea	0.00248888593748454	0.0151298066199718	0	0.000568494882728101
k__Bacteria;p__Bacteroidetes;c__Bacteroidia;o__Bacteroidales;__;__	0.00268054637031222	0.0157165768645310	0.00200788030217186	0.00750897128197941

k__Bacteria;p__Firmicutes;c__Clostridia;o__Clostridiales;f__Veillonellaceae;g__	0.00272148517134736	0.015716576864531	0.0000369318181818182	0.000403571955112187
k__Bacteria;p__Firmicutes;c__Clostridia;o__Clostridiales;f__Lachnospiraceae;g__Oribacterium	0.00284273529577778	0.0160163866664553	0.000532425830292538	0.00163017724927436
k__Bacteria;p__Bacteroidetes;c__Bacteroidia;o__Bacteroidales;f__Porphyromonadaceae;g__Tannerella	0.00336466432149853	0.0185056537682419	0.0305468604540926	0.053468457860018
k__Bacteria;p__Bacteroidetes;c__Sphingobacteriia;o__Sphingobacteriales;f__Sphingobacteriaceae;g__Pedobacter	0.00514546601452188	0.0276419220780129	0.000375003280478809	0
k__Bacteria;p__Firmicutes;c__Clostridia;o__Clostridiales;f__Ruminococcaceae;g__Ruminococcus	0.00878830975425357	0.0454615274049267	0.00317256043083474	0.0000761432190314789
k__Bacteria;p__Firmicutes;c__Clostridia;o__Clostridiales;f__Lachnospiraceae;g__Lachnoanaerobaculum	0.00885614170225845	0.0454615274049267	0.000405523305298843	0.00169209084796847
k__Bacteria;p__Proteobacteria;c__Gammaproteobacteria;o__Alteromonadales;f__[Chromatiaceae];g__Alishewanella	0.00996874092801338	0.0492167845035	0.00667985459932246	0.000445904062855519
k__Bacteria;p__Firmicutes;c__Clostridia;o__Clostridiales;f__Clostridiaceae;g__Clostridium	0.0100748513880316	0.0492167845035	0.00563765931454027	0.000608739738728708
k__Bacteria;p__Fusobacteria;c__Fusobacteriia;o__Fusobacteriales;f__Leptotrichiaceae;g__Leptotrichia	0.0103298131685447	0.0492167845035	0.0021245582723576	0.0109927700858195
k__Bacteria;p__Proteobacteria;c__Epsilonproteobacteria;o__Campylobacterales;f__Helicobacteraceae;g__Wolinella	0.0104399239855909	0.0492167845035	0	0.000114850640606329
k__Bacteria;p__Firmicutes;c__Clostridia;o__Clostridiales;f__Lachnospiraceae;g__Johnsonella	0.0115558775330482	0.0531421577401816	0.00414128011113663	0.0093872564656937
k__Bacteria;p__Bacteroidetes;c__Sphingobacteriia;o__Sphingobacteriales;f__Sphingobacteriaceae;g__Sphingobacterium	0.0117326841764037	0.0531421577401816	0.00253508405254652	0.000388125516800053

k__Bacteria;p__Proteobacteria;c__Alphaproteobacteria;o__Sphingomonadales;f__Sphingomonadaceae;g__Novosphingobium	0.0122425901229375	0.0543853522768955	0.0163768680468491	0.00203760467201337
k__Bacteria;p__Proteobacteria;c__Gammaproteobacteria;o__Aeromonadales;f__Aeromonadaceae;g__Aeromonas	0.0126100379859645	0.0549607315992037	0.0115720285190774	0.00110431330015511
k__Bacteria;p__Actinobacteria;c__Coriobacteriia;o__Coriobacteriales;f__Coriobacteriaceae;g__Atopobium	0.0170705259149819	0.0730239164140893	0.00078647705018408	0.00318931188189061
k__Bacteria;p__Firmicutes;c__Bacilli;o__Bacillales;f__Paenibacillaceae;g__Paenibacillus	0.0184953149625019	0.077680322842508	0.00338232900746662	0.000443831821522706
k__Bacteria;p__Bacteroidetes;c__Flavobacteriia;o__Flavobacteriales;f__[Weeksellaceae];g__Chryseobacterium	0.0195039975247131	0.0804539897894417	0.00617603368121491	0.000395135508040151
k__Bacteria;p__Proteobacteria;c__Alphaproteobacteria;o__Rhizobiales;f__Rhizobiaceae;__	0.0208570245951454	0.0830684945082516	0.00193611495887619	0
k__Bacteria;p__Synergistetes;c__Synergistia;o__Synergistales;f__Dethiosulfovibrionaceae;g__Pyramidobacter	0.0208570245951454	0.0830684945082516	0	0.00244697331439855
k__Bacteria;p__Proteobacteria;c__Deltaproteobacteria;o__Desulfovibrionales;f__Desulfovibrionaceae;g__Desulfovibrio	0.0219532861781437	0.0859526967313761	0.000319786664016445	0.00549725558615802

Table S1B. Relative abundance of the most significant genera in the samples collected in periodontitis sites and healthy sites as assessed by Mann-Whitney U tests with false-discovery-rate correction.

**Feature**	**P value**	**FDR corrected P value**
c6fe05e0eadde34514f9c0114e4e6217k__Bacteria; p__Actinobacteria; c__Actinobacteria; o__Actinomycetales; f__Actinomycetaceae; g__Actinomyces; s__	0.000000674390143846614	0.000923272939967511
bc5ba73185ee3b14bb2d58b32352573dk__Bacteria; p__Firmicutes; c__Clostridia; o__Clostridiales; f__Veillonellaceae; g__Dialister; s__	0.00000101570180414468	0.000923272939967511

551afecd8c6458bbc62c7b8c202333d6k__Bacteria; p__Spirochaetes; c__Spirochaetes; o__Spirochaetales; f__Spirochaetaceae; g__Treponema; s__	0.00000400970689334508	0.00228806082949444
73185b4ea26a363e443916df0e80da85k__Bacteria; p__Synergistetes; c__Synergistia; o__Synergistales; f__Dethiosulfovibrionaceae; g__TG5; s__	0.00000918614206296093	0.00228806082949444
c95baa0e17797184c2babd17b5b602fdk__Bacteria; p__Bacteroidetes; c__Bacteroidia; o__Bacteroidales; f__; g__; s__	0.00000947382080970403	0.00228806082949444
f872dd32faa2d1ce555803411d38bd04k__Bacteria; p__Synergistetes; c__Synergistia; o__Synergistales; f__Dethiosulfovibrionaceae; g__TG5; s__	0.0000096820260747471	0.00228806082949444
d408960aaa7ed992b378f7b989c83352k__Bacteria; p__Spirochaetes; c__Spirochaetes; o__Spirochaetales; f__Spirochaetaceae; g__Treponema; s__socranskii	0.0000110861296220094	0.00228806082949444
1b84689077164dbfd545d0166414c410k__Bacteria; p__Firmicutes; c__Bacilli; o__Lactobacillales; f__Enterococcaceae; g__Enterococcus	0.0000129923356289357	0.00228806082949444
f37c1e77018a107cdfb67494ad642c71k__Bacteria; p__Spirochaetes; c__Spirochaetes; o__Spirochaetales; f__Spirochaetaceae; g__Treponema; s__	0.0000132835669621462	0.00228806082949444
83e17f478f7b8555164930da9d203a1bk__Bacteria; p__Actinobacteria; c__Actinobacteria; o__Actinomycetales; f__Actinomycetaceae; g__Actinomyces; s__	0.0000133431793534294	0.00228806082949444
9564ad66d1862a76320e4f0f0611b951k__Bacteria; p__Spirochaetes; c__Spirochaetes; o__Spirochaetales; f__Spirochaetaceae; g__Treponema; s__socranskii	0.0000138441524336848	0.00228806082949444
cfe787fd73e7e99342f3795b93c8094ek__Bacteria; p__Spirochaetes; c__Spirochaetes; o__Spirochaetales; f__Spirochaetaceae; g__Treponema; s__	0.0000171038818620973	0.00259123810210775
76c67adc9e35b61b046958ca9265cb52k__Bacteria; p__Firmicutes; c__Clostridia; o__Clostridiales; f__Veillonellaceae; g__Megasphaera; s__	0.0000217572629266551	0.00304266953851223
e96068176ed806770bce75b445f52bf0k__Bacteria; p__Firmicutes; c__Clostridia; o__Clostridiales; f__Veillonellaceae; g__Dialister; s__	0.0000302888796290739	0.00384817726949115

144f6dc6ca0a9419a0d4441bd06fded6k__Bacteria; p__Firmicutes; c__Clostridia; o__Clostridiales; f__[Mogibacteriaceae]; g__; s__	0.0000317506375370557	0.00384817726949115
13799ebd90b035f0f87a6cfb4b5b9785k__Bacteria; p__Spirochaetes; c__Spirochaetes; o__Spirochaetales; f__Spirochaetaceae; g__Treponema; s__	0.0000381319770916692	0.0042425011190912
ac8d1d95775840e7164322beaad96fc3k__Bacteria; p__Firmicutes; c__Clostridia; o__Clostridiales; f__Veillonellaceae; g__Schwartzia; s__	0.0000416942741784366	0.0042425011190912
790fe3b27ec763bb3d21d47ab02eca80k__Bacteria; p__Synergistetes; c__Synergistia; o__Synergistales; f__Dethiosulfovibrionaceae; g__TG5; s__	0.0000442632153632012	0.0042425011190912
d4787d376913d382877e5f423dc6fcbfk__Bacteria; p__Synergistetes; c__Synergistia; o__Synergistales; f__Dethiosulfovibrionaceae; g__TG5; s__	0.0000443385705515581	0.0042425011190912
4791c48746fd0d53a6252ab17a4bf90fk__Bacteria; p__Bacteroidetes; c__Bacteroidia; o__Bacteroidales; f__; g__; s__	0.0000549134353812854	0.00499163127615884
36839726526cc2fd509378431a87de2ek__Bacteria; p__TM7; c__TM7-3; o__I025; f__Rs-045; g__; s__	0.0000615730442498487	0.00533046640220119
2b18cb213d6dc1f56502ae84e29b4587k__Bacteria; p__Bacteroidetes; c__Bacteroidia; o__Bacteroidales; f__; g__; s__	0.0000765085487379435	0.00632238825479915
5d23c90c9c9e913dbeeb783b3a965744k__Bacteria; p__Firmicutes; c__Clostridia; o__Clostridiales; f__Veillonellaceae; g__Selenomonas; s__	0.0000911546603150587	0.00720518141099029
a9dbae538a72ec4456d0c44a3311c9c7k__Bacteria; p__Firmicutes; c__Clostridia; o__Clostridiales; f__Veillonellaceae; g__Megasphaera; s__	0.000104728900671664	0.00758945151231151
4a2c4912f078689e3a3f86781668c24bk__Bacteria; p__TM7; c__TM7-3; o__I025; f__Rs-045; g__; s__	0.000107244448240939	0.00758945151231151
2a0cc23acd500009cb08f6e1e585cec5k__Bacteria; p__Proteobacteria; c__Deltaproteobacteria; o__Desulfobacterales; f__Desulfobulbaceae; g__Desulfobulbus; s__	0.000111161089824584	0.00758945151231151
519cc5eed90752b5ec6f4bf826414be9k__Bacteria; p__TM7; c__TM7-3; o__; f__; g__; s__	0.000112714626420468	0.00758945151231151
5a1ce3b38295597fa289c6d4a87dc931k__Bacteria; p__Proteobacteria; c__Epsilonproteobacteria; o__Campylobacterales; f__Campylobacteraceae; g__Campylobacter; s__rectus	0.000120306407578308	0.00781132317776301

2bbe980ffbf03d99af48e37a96b846d2k__Bacteria; p__Bacteroidetes; c__Bacteroidia; o__Bacteroidales; f__; g__; s__	0.000128731287721336	0.00807012003715134
bf52686b796569563cd1101d06a81652k__Bacteria; p__Proteobacteria; c__Epsilonproteobacteria; o__Campylobacterales; f__Campylobacteraceae; g__Campylobacter; s__rectus	0.000146463719180786	0.00874760141359196
c857267853fcb382f31c347a4cd18779k__Bacteria; p__TM7; c__TM7-3; o__; f__; g__; s__	0.000149161520253768	0.00874760141359196
a8ccf8547a9588b16888ed0c2965372dk__Bacteria; p__Firmicutes; c__Bacilli; o__Lactobacillales; f__Enterococcaceae; g__Enterococcus	0.000181665400033847	0.010038407749674
9aefcdd447c21a7e8db1289570b0525ak__Bacteria; p__Proteobacteria; c__Deltaproteobacteria; o__Desulfobacterales; f__Desulfobulbaceae; g__Desulfobulbus; s__	0.000182499703681771	0.010038407749674
f21b8cc7513de3a8858f616d95aa0ef0k__Bacteria; p__Actinobacteria; c__Actinobacteria; o__Actinomycetales; f__Actinomycetaceae; g__Actinomyces; s__	0.000187736998618766	0.010038407749674
69356212695e27fe4c2cf626f17d383ek__Bacteria; p__Firmicutes; c__Clostridia; o__Clostridiales; f__Eubacteriaceae; g__Pseudoramibacter_Eubacterium; s__	0.000195668063425801	0.0101635582659459
a329e9b559532fe253c6ec195ad9bae9k__Bacteria; p__Firmicutes; c__Clostridia; o__Clostridiales; f__Veillonellaceae; g__Schwartzia; s__	0.000204925784795082	0.0103487521321517
2b0da9fcbb02a4e7e60c5789761a8e43k__Bacteria; p__Actinobacteria; c__Coriobacteriia; o__Coriobacteriales; f__Coriobacteriaceae	0.000213921317448761	0.010511052841131
ecdd86b0cb840f683461c6bc18db6ddak__Bacteria; p__Firmicutes; c__Clostridia; o__Clostridiales; f__Veillonellaceae; g__Schwartzia; s__	0.000252949905360099	0.0118945462630402
bf3651ee44868000213c7da680830495k__Bacteria; p__Firmicutes; c__Clostridia; o__Clostridiales; f__Veillonellaceae; g__Selenomonas; s__	0.000255163533695581	0.0118945462630402
d45149d997d8a052551e26cf9a001017k__Bacteria; p__Firmicutes; c__Clostridia; o__Clostridiales; f__Peptostreptococcaceae; g__Filifactor; s__	0.000265889676595768	0.0120846858012776
9ac732c08f0112c91f65d27aad0d79a8k__Bacteria; p__Firmicutes; c__Clostridia; o__Clostridiales; f__Eubacteriaceae; g__Pseudoramibacter_Eubacterium; s__	0.000280867388149132	0.0124540710159786

a054733fa78e1f68418eb5c25bba3f3ck__Bacteria; p__Chloroflexi; c__Anaerolineae; o__Anaerolineales; f__Anaerolinaceae; g__SHD-231; s__	0.0003016912358484	0.0130589206374379
8e0097c236b4dcfd96c4f15431283011k__Bacteria; p__Chloroflexi; c__Anaerolineae; o__Anaerolineales; f__Anaerolinaceae; g__SHD-231; s__	0.000315110505774773	0.0133225790581055
a49fd8e92bf0c535490f157c5830220dk__Bacteria; p__Firmicutes; c__Clostridia; o__Clostridiales; f__Peptostreptococcaceae; g__Filifactor; s__	0.000342508115434604	0.0141518125877298
5c3bed3239c2712137b24fe8d30f54e3k__Bacteria; p__Fusobacteria; c__Fusobacteriia; o__Fusobacteriales; f__Fusobacteriaceae; g__Fusobacterium; s__	0.000360530712824447	0.0145023034892892
8d0a25543b5540681c5fa5a24b788d2ck__Bacteria; p__Firmicutes; c__Clostridia; o__Clostridiales; f__Veillonellaceae; g__Selenomonas; s__	0.000367302319523396	0.0145023034892892
fe83ceb71e65a2e803c46db3f91c40dbk__Bacteria; p__Fusobacteria; c__Fusobacteriia; o__Fusobacteriales; f__Fusobacteriaceae; g__Fusobacterium; s__	0.000374922037401866	0.0145023034892892
a79e88579e498b17533d0eb3fbc97097k__Bacteria; p__Actinobacteria; c__Coriobacteriia; o__Coriobacteriales; f__Coriobacteriaceae	0.00044095899500005	0.0167013219356269
0914f9d58849b13a1b0e58a9a5aedb52k__Bacteria; p__Firmicutes; c__Clostridia; o__Clostridiales; f__[Mogibacteriaceae]; g__; s__	0.000546275022207647	0.0200832315301099
3be1ebe170fe290f25d50b19bd38e5a3k__Bacteria; p__Firmicutes; c__Clostridia; o__Clostridiales; f__[Mogibacteriaceae]; g__; s__	0.00055234410148817	0.0200832315301099
605e49a560632564826705835a96340ek__Bacteria; p__Firmicutes; c__Clostridia; o__Clostridiales; f__[Acidaminobacteraceae]; g__; s__	0.000573818328586224	0.0204549357131325
1c76fdf12e1bc025eb1fd181502e26f8k__Bacteria; p__Firmicutes; c__Clostridia; o__Clostridiales; f__Ruminococcaceae; g__Ethanoligenens; s__	0.000591805641901514	0.0206904357110952
5cccbe2d225183bcf86c8550bf6e5663k__Bacteria; p__Spirochaetes; c__Spirochaetes; o__Spirochaetales; f__Spirochaetaceae; g__Treponema; s__	0.000723300540975533	0.0248105732734626

817f2e9e3d43576ce1ef9a949941568bk__Bacteria; p__Firmicutes; c__Clostridia; o__Clostridiales; f__Lachnospiraceae; g__Moryella; s__	0.000914380159238831	0.0307841320277073
a8272bacf2c2f52cf6d6671945f3b8b1k__Bacteria; p__Firmicutes; c__Clostridia; o__Clostridiales; f__[Mogibacteriaceae]; g__Mogibacterium; s__	0.000959971963750901	0.0317314369108934
3215553ee75c244878f20d87fac2eec4k__Bacteria; p__Firmicutes; c__Clostridia; o__Clostridiales; f__Veillonellaceae; g__Selenomonas; s__	0.00100993022971941	0.0327866635291052
fd1bf824f51935de4165b38b65de449fk__Bacteria; p__Actinobacteria; c__Coriobacteriia; o__Coriobacteriales; f__Coriobacteriaceae; g__Olsenella; s__uli	0.00107495521383502	0.0342854136623169
c35f25a0fbe693b2756952031d032a19k__Bacteria; p__Bacteroidetes; c__Bacteroidia; o__Bacteroidales; f__Porphyromonadaceae; g__Porphyromonas; s__endodontalis	0.00114102386582525	0.0357651963460398
eb1b52c186280f228ba2ad84174e2375k__Bacteria; p__SR1; c__; o__; f__; g__; s__	0.00117703007848985	0.0362684861473651
7d9a6febbb1e3dbaf757b457b35793c8k__Bacteria; p__Actinobacteria; c__Actinobacteria; o__Actinomycetales; f__Actinomycetaceae; g__Actinomyces; s__	0.00129524337337938	0.0392458742133951
cea3087aa1309826968d10332c43cac2k__Bacteria; p__Actinobacteria; c__Coriobacteriia; o__Coriobacteriales; f__Coriobacteriaceae; g__Slackia; s__	0.00133493631442647	0.0397854790102841
adb88e338556ef8f64fc2274be474a2bk__Bacteria; p__Proteobacteria; c__Gammaproteobacteria; o__Aeromonadales; f__Aeromonadaceae; g__Aeromonas	0.00140078505921287	0.0410746328653064
f6417b4f09f1d0a99a0f7e3b99b633b6k__Bacteria; p__Firmicutes; c__Clostridia; o__Clostridiales; f__Veillonellaceae; g__Selenomonas; s__	0.00154894107704419	0.0444766367766848
1566fb11e14dfd6eb34380ea013681b8k__Bacteria; p__Bacteroidetes; c__Bacteroidia; o__Bacteroidales; f__[Paraprevotellaceae]; g__[Prevotella]; s__tannerae	0.0015657341879581	0.0444766367766848
d3ca912e1fa90614ab126ec80ce08bffk__Bacteria; p__Actinobacteria; c__Coriobacteriia; o__Coriobacteriales; f__Coriobacteriaceae; g__Olsenella; s__uli	0.00168808453265172	0.0472144258517049
5150194a19241bd02c775d7141db7a3fk__Bacteria; p__Firmicutes; c__Clostridia; o__Clostridiales; f__[Mogibacteriaceae]; g__; s__	0.00177050160418882	0.0487692714608374

cefd913c459ceb525dea51c11fed8410k__Bacteria; p__Bacteroidetes; c__Bacteroidia; o__Bacteroidales; f__Prevotellaceae; g__Prevotella; s__nigrescens	0.00187595297839018	0.0504736793925543
0e186d0679dd22f725dae36e97f1a419k__Bacteria; p__Firmicutes; c__Clostridia; o__Clostridiales; f__[Mogibacteriaceae]; g__; s__	0.0018879043997215	0.0504736793925543
09b40fa2d19acbe34df3ac8ce140197dk__Bacteria; p__Bacteroidetes; c__Bacteroidia; o__Bacteroidales; f__Porphyromonadaceae; g__Porphyromonas; s__endodontalis	0.00195211154532687	0.0514338954986122
cd719f1457b504943146deec30dfa854k__Bacteria; p__Spirochaetes; c__Spirochaetes; o__Spirochaetales; f__Spirochaetaceae; g__Treponema; s__	0.00201376394133013	0.052300326361974
daa0a485041ae3854efb3e3504688f04k__Bacteria; p__Bacteroidetes; c__Bacteroidia; o__Bacteroidales	0.00212724557153839	0.0537309372918934
d4a306d7fca5f9e6600812d9885a8bc5k__Bacteria; p__Firmicutes; c__Clostridia; o__Clostridiales; f__Veillonellaceae; g__Schwartzia; s__	0.00212795791255023	0.0537309372918934
5e0c5fa206b7fd068d72d24ea5d3c1f8k__Bacteria; p__Firmicutes; c__Clostridia; o__Clostridiales; f__Peptostreptococcaceae; g__Peptostreptococcus; s__	0.00223194623139672	0.0552842652974155
8aec1d202d5fef48dcec9784f1fdeb61k__Bacteria; p__Firmicutes; c__Clostridia; o__Clostridiales; f__Veillonellaceae; g__Dialister; s__	0.00225029462706752	0.0552842652974155
24b9b343cc35188986de7591182a1e82k__Bacteria; p__Firmicutes; c__Clostridia; o__Clostridiales; f__Lachnospiraceae; g__Moryella; s__	0.00236679140379998	0.055828747033421
9fe5a73817c09ab0936b15f4a9a9e754k__Bacteria; p__Actinobacteria; c__Actinobacteria; o__Actinomycetales; f__Propionibacteriaceae; g__Propionibacterium; s__	0.0024027594952735	0.055828747033421
caf53c77c1727cd43a2d4425aa7e8149k__Bacteria; p__Bacteroidetes; c__Bacteroidia; o__Bacteroidales; f__Porphyromonadaceae; g__Tannerella; s__	0.00242161525786669	0.055828747033421
ec6c428af11dff47772e6d9f5e96194dk__Bacteria; p__Firmicutes; c__Clostridia; o__Clostridiales; f__[Mogibacteriaceae]; g__; s__	0.00245965657343451	0.055828747033421
dfe38c58277aea481171bfe846e3c561k__Bacteria; p__Fusobacteria; c__Fusobacteriia; o__Fusobacteriales; f__Fusobacteriaceae; g__Fusobacterium; s__	0.00247716869537676	0.055828747033421

03c934fdf5c5364a7cd07168f0f14d23k__Bacteria; p__Firmicutes; c__Clostridia; o__Clostridiales; f__[Mogibacteriaceae]; g__; s__	0.00248155867595045	0.055828747033421
4f488ca2f85e46a8cb5f1ff6dbcf4acbk__Bacteria; p__Fusobacteria; c__Fusobacteriia; o__Fusobacteriales; f__Leptotrichiaceae; g__Leptotrichia; s__	0.00248741942228114	0.055828747033421
df0fd01e72ffe52482b59a99681dfb89k__Bacteria; p__Bacteroidetes; c__Bacteroidia; o__Bacteroidales; f__Prevotellaceae; g__Prevotella; s__	0.00302033204912169	0.0669629715280883
6d338377a30786cb18feca9a0559ee19k__Bacteria; p__Proteobacteria; c__Gammaproteobacteria; o__Enterobacteriales; f__Enterobacteriaceae	0.00317538228601869	0.0695523493491804
2bed993692d7a0d8d6b66cd44a260b05k__Bacteria; p__Bacteroidetes; c__Bacteroidia; o__Bacteroidales; f__Prevotellaceae; g__Prevotella; s__nigrescens	0.00322677132494882	0.0698365508185351
e078ee117330ac196ca272bf18b08f8ck__Bacteria; p__Firmicutes; c__Bacilli; o__Lactobacillales	0.00346824508287025	0.0741796418900955
3a804f03bb646fdbf7e0573543523525k__Bacteria; p__Actinobacteria; c__Actinobacteria; o__Actinomycetales; f__Actinomycetaceae; g__Actinomyces; s__	0.00396001607733144	0.0837128980068438
d4418cb8173f9aa6b7728ef28f2d19a0k__Bacteria; p__Firmicutes; c__Bacilli; o__Lactobacillales	0.00418508846226731	0.0874539175218616
65e7acabb644a37d980ca633c1c26e7dk__Bacteria; p__Firmicutes; c__Clostridia; o__Clostridiales; f__[Mogibacteriaceae]; g__Mogibacterium; s__	0.00450842840851339	0.0931400323486062
cf2fa22dccc7fb6803c8159d8a1238e4k__Bacteria; p__Actinobacteria; c__Actinobacteria; o__Actinomycetales; f__Actinomycetaceae; g__Actinomyces; s__hyovaginalis	0.00474796962279649	0.0935128324097611
70a6351e6aebc07f4687dbd9f6042625k__Bacteria; p__Firmicutes; c__Bacilli; o__Bacillales; f__[Exiguobacteraceae]; g__; s__	0.00488784208319431	0.0935128324097611
8f966d7ebee98150bcde5873000e1121k__Bacteria; p__Bacteroidetes; c__Bacteroidia; o__Bacteroidales; f__Porphyromonadaceae; g__Tannerella; s__	0.00507718686512873	0.0935128324097611
b5d801665b8a57b7ec8e6d692022f21fk__Bacteria; p__Firmicutes; c__Bacilli; o__Lactobacillales; f__Enterococcaceae; g__Enterococcus	0.00513371591998184	0.0935128324097611

446b8b9418dd980c65b0193e336d7397k__Bacteria; p__Firmicutes; c__Erysipelotrichi; o__Erysipelotrichales; f__Erysipelotrichaceae; g__Sharpea; s__	0.00513958909533904	0.0935128324097611
388a7ccbe8686fab8f272eac3b2fc879k__Bacteria; p__Firmicutes; c__Bacilli; o__Lactobacillales; f__Streptococcaceae; g__Lactococcus; s__	0.00513958909533904	0.0935128324097611
430c12c4272206d85e53713539a8acf4k__Bacteria; p__Firmicutes; c__Clostridia; o__Clostridiales; f__Veillonellaceae; g__Selenomonas; s__	0.005142527086965	0.0935128324097611
41225c8338f999f75b73968518653579k__Bacteria; p__Fusobacteria; c__Fusobacteriia; o__Fusobacteriales; f__Leptotrichiaceae; g__Leptotrichia; s__	0.005142527086965	0.0935128324097611
1392616d0058071c29c79221e0ecdb90k__Bacteria; p__Bacteroidetes; c__Bacteroidia; o__Bacteroidales; f__Prevotellaceae; g__Prevotella; s__	0.005142527086965	0.0935128324097611
73d339dcc87bbf534c6c9c0592422680k__Bacteria; p__Bacteroidetes; c__Bacteroidia; o__Bacteroidales; f__; g__; s__	0.005142527086965	0.0935128324097611
a6abe48ab079a4d35a15c59017217bd6k__Bacteria; p__Fusobacteria; c__Fusobacteriia; o__Fusobacteriales; f__Leptotrichiaceae; g__Leptotrichia; s__	0.005142527086965	0.0935128324097611
b59bd58bd9c78ace8f946f119363b459k__Bacteria; p__SR1; c__; o__; f__; g__; s__	0.00514546601452188	0.0935128324097611
a68bacb3b6b8130e0258cb628daeee22k__Bacteria; p__Bacteroidetes; c__Bacteroidia; o__Bacteroidales; f__Porphyromonadaceae; g__Tannerella; s__	0.00519515735609784	0.0935128324097611
c3d7a43c6666766f50d4962db19f38e7k__Bacteria; p__Bacteroidetes; c__Bacteroidia; o__Bacteroidales; f__Porphyromonadaceae; g__Paludibacter; s__	0.00525406572337695	0.0936459949519539
855a533637bcdd353eb10c47cb021d47k__Bacteria; p__Firmicutes; c__Clostridia; o__Clostridiales; f__[Acidaminobacteraceae]; g__; s__	0.00530968711219886	0.0937185550483256
e8c5d0c8c25d7174965a425152d8ec9bk__Bacteria; p__Bacteroidetes; c__Bacteroidia; o__Bacteroidales; f__Prevotellaceae; g__Prevotella; s__	0.00547173019039772	0.0951454983462067
a947d19dd35a6557cc57c114c2d88f6dk__Bacteria; p__Bacteroidetes; c__Bacteroidia; o__Bacteroidales; f__[Paraprevotellaceae]; g__[Prevotella]; s__tannerae	0.00549520204969841	0.0951454983462067

Table S1C. Relative abundance of the most significant ASV in the samples collected in periodontitis sites and healthy sites as assessed by Mann-Whitney U tests with false-discovery-rate correction.

**Feature**	**P value**	**FDR corrected P value**	**mean relative abundance healthy sites**
K01885	0.0000000399649528799859	0.000217329225747356	0.000634224634696454
K02493	0.0000000497502187039539	0.000217329225747356	0.000724771164667135
K09141	0.0000000618408116515288	0.000217329225747356	0.0000988783440930178
K16887	0.000000161650784712705	0.000221432691509016	0.0000837155697961101
K00537	0.000000199327343052566	0.000221432691509016	0.000525176425315405
K11180	0.000000199327343052566	0.000221432691509016	0.0000538596040734554
K11181	0.000000199327343052566	0.000221432691509016	0.0000536093232750798
K05772	0.000000245426793896234	0.000221432691509016	0.000042399115942591
K03388	0.000000272183543826178	0.000221432691509016	0.0000513825081518665
K19116	0.000000272183543826178	0.000221432691509016	0.0000268414419934178
K04070	0.000000370451440882709	0.000221432691509016	0.0000982727445737541
K05773	0.000000370451440882709	0.000221432691509016	0.0000309734365091087
K11782	0.000000370451440882709	0.000221432691509016	0.0000316696425131894
K01922	0.000000410239797767579	0.000221432691509016	0.000139914230566253
K05299	0.000000454136099359966	0.000221432691509016	0.0000254651382345501
K06857	0.000000454136099359966	0.000221432691509016	0.0000290082147274849
K11785	0.000000454136099359966	0.000221432691509016	0.0000221902762310504
K05362	0.000000502546242123424	0.000221432691509016	0.000137951425848812
K11784	0.000000502546242123424	0.000221432691509016	0.0000331599923787385
K09740	0.00000055591428809767	0.000221432691509016	0.0000798772987881901
K16885	0.00000055591428809767	0.000221432691509016	0.0000649002179551631

K03686	0.000000614725854433303	0.000221432691509016	0.0008653370528741
K19419	0.000000614725854433303	0.000221432691509016	0.0000688338225919987
K07404	0.000000679511784310068	0.000221432691509016	0.000214231545047507
K08253	0.000000679511784310068	0.000221432691509016	0.0000479068484855994
K00958	0.000000829380406221231	0.000221432691509016	0.0000355150320958098
K01155	0.000000829380406221231	0.000221432691509016	0.000185955825838931
K02236	0.000000829380406221231	0.000221432691509016	0.00016689777433406
K04077	0.000000829380406221231	0.000221432691509016	0.000797226355907243
K06878	0.000000829380406221231	0.000221432691509016	0.000250837750782942
K07714	0.000000829380406221231	0.000221432691509016	0.0000265633925917191
K03739	0.000000915788332803924	0.000221432691509016	0.000160659868611631
K03740	0.000000915788332803924	0.000221432691509016	0.000161027244416888
K18285	0.000000915788332803924	0.000221432691509016	0.0000429322945767112
K02428	0.00000101083076871153	0.000221432691509016	0.000842237230990787
K03465	0.00000101083076871153	0.000221432691509016	0.000120084822140722
K03933	0.00000101083076871153	0.000221432691509016	0.000070902504465135
K07707	0.00000101083076871153	0.000221432691509016	0.000224329528068607
K16886	0.00000101083076871153	0.000221432691509016	0.00000689897428396655
K00687	0.000001115331189493	0.000221432691509016	0.000131178439329202
K16509	0.000001115331189493	0.000221432691509016	0.00037804819251603
K19710	0.000001115331189493	0.000221432691509016	0.0000857734418361348
K00383	0.00000123018754345587	0.000221432691509016	0.000247106088163256
K02440	0.00000123018754345587	0.000221432691509016	0.000460578997131037
K05593	0.00000123018754345587	0.000221432691509016	0.000119687812289722
K07467	0.00000123018754345587	0.000221432691509016	0.000246051773452677

K12556	0.00000123018754345587	0.000221432691509016	0.000129370769158944
K13929	0.00000123018754345587	0.000221432691509016	0.0000705607513162033
K00105	0.00000135637858311105	0.000221432691509016	0.000123944049011046
K02082	0.00000135637858311105	0.000221432691509016	0.000105441729170464
K03697	0.00000135637858311105	0.000221432691509016	0.000125655168874951
K08982	0.00000135637858311105	0.000221432691509016	0.0000290986054794242
K11706	0.00000135637858311105	0.000221432691509016	0.000173865052712788
K13038	0.00000135637858311105	0.000221432691509016	0.000566034037628397
K13932	0.00000135637858311105	0.000221432691509016	0.000070551422586573
K02008	0.00000149497069640244	0.000221432691509016	0.00012413312264324
K02779	0.00000149497069640244	0.000221432691509016	0.000210151016685277
K03346	0.00000149497069640244	0.000221432691509016	0.000155545241516348
K03367	0.00000149497069640244	0.000221432691509016	0.000167386518347016
K03930	0.00000149497069640244	0.000221432691509016	0.00013070266246237
K06198	0.00000149497069640244	0.000221432691509016	0.0001340217136928
K09116	0.00000149497069640244	0.000221432691509016	0.000011465004234231
K13930	0.00000149497069640244	0.000221432691509016	0.0000688822644991053
K13934	0.00000149497069640244	0.000221432691509016	0.0000703352069383497
K14188	0.00000149497069640244	0.000221432691509016	0.000163766911345833
K19115	0.00000149497069640244	0.000221432691509016	0.000030936585683141
K03095	0.00000164712527365909	0.000221432691509016	0.000121767806338998
K07660	0.00000164712527365909	0.000221432691509016	0.0000418933060031735
K10013	0.00000164712527365909	0.000221432691509016	0.0000179433266251694
K13931	0.00000164712527365909	0.000221432691509016	0.0000702239477037776
K15342	0.00000164712527365909	0.000221432691509016	0.000485859262751768

K03825	0.00000181410664848461	0.000221432691509016	0.0000341198303740731
K06990	0.00000181410664848461	0.000221432691509016	0.00004681579704887
K11704	0.00000181410664848461	0.000221432691509016	0.000174969588977242
K12525	0.00000181410664848461	0.000221432691509016	0.0000223299820146314
K18237	0.00000181410664848461	0.000221432691509016	0.0000012342130391443
K00762	0.00000199729065319521	0.000221432691509016	0.000710986907146674
K01484	0.00000199729065319521	0.000221432691509016	0.0000507888826998622
K02819	0.00000199729065319521	0.000221432691509016	0.000181366184723605
K03389	0.00000199729065319521	0.000221432691509016	0.0000362234729353292
K03564	0.00000199729065319521	0.000221432691509016	0.000417682317216159
K06286	0.00000199729065319521	0.000221432691509016	0.000141696387831016
K07533	0.00000199729065319521	0.000221432691509016	0.000262179955413911
K13935	0.00000199729065319521	0.000221432691509016	0.0000616178217408233
K15723	0.00000199729065319521	0.000221432691509016	0.0000221212956593237
K19005	0.00000199729065319521	0.000221432691509016	0.000173951700630612
K00395	0.00000219817383193646	0.000221432691509016	0.0000218333752598138
K00486	0.00000219817383193646	0.000221432691509016	0.0000113240452980504
K00800	0.00000219817383193646	0.000221432691509016	0.000712836635130302
K01215	0.00000219817383193646	0.000221432691509016	0.000153412927819654
K03184	0.00000219817383193646	0.000221432691509016	0.0000319562390178917
K03531	0.00000219817383193646	0.000221432691509016	0.000753254546414493
K07136	0.00000219817383193646	0.000221432691509016	0.0000422606081954548
K07146	0.00000219817383193646	0.000221432691509016	0.000272500127677366
K07345	0.00000219817383193646	0.000221432691509016	0.0000526643309434323
K11085	0.00000219817383193646	0.000221432691509016	0.000454525192367247

K01261	0.00000241838335725486	0.000221432691509016	0.000140204738454209
K01844	0.00000241838335725486	0.000221432691509016	0.0000646222545065438
K02086	0.00000241838335725486	0.000221432691509016	0.000143669586285372
K05910	0.00000241838335725486	0.000221432691509016	0.0000660184919838832
K07070	0.00000241838335725486	0.000221432691509016	0.0000318380543479259
K07706	0.00000241838335725486	0.000221432691509016	0.000203367977421999
K12293	0.00000241838335725486	0.000221432691509016	0.0000456787060642847
K13057	0.00000241838335725486	0.000221432691509016	0.0000338236503877647
K16169	0.00000241838335725486	0.000221432691509016	0.000139598652582366
K16693	0.00000241838335725486	0.000221432691509016	0.00000605614942943825
K18011	0.00000241838335725486	0.000221432691509016	0.0000646927101520438
K10461	0.00000265822105024469	0.000221432691509016	0.000000619148521904013
K00864	0.00000265968769867704	0.000221432691509016	0.000527740032125822
K01146	0.00000265968769867704	0.000221432691509016	0.0000210663748555657
K02248	0.00000265968769867704	0.000221432691509016	0.0000950209182044395
K02853	0.00000265968769867704	0.000221432691509016	0.00000581160401234306
K03761	0.00000265968769867704	0.000221432691509016	0.0000631019930908956
K05368	0.00000265968769867704	0.000221432691509016	0.0000210667026246272
K06169	0.00000265968769867704	0.000221432691509016	0.0000428975647412542
K06858	0.00000265968769867704	0.000221432691509016	0.0000281048541657316
K08351	0.00000265968769867704	0.000221432691509016	0.0000184561590858417
K12292	0.00000265968769867704	0.000221432691509016	0.0000488083430507733
K12582	0.00000265968769867704	0.000221432691509016	0.00000581008052617097
K12961	0.00000265968769867704	0.000221432691509016	0.0000210664027534748
K17999	0.0000029224084863488	0.000221432691509016	0.000000372880151436163

K00394	0.00000292400809476094	0.000221432691509016	0.0000224924263697255
K01598	0.00000292400809476094	0.000221432691509016	0.000146102660748014
K02043	0.00000292400809476094	0.000221432691509016	0.0000275542987424163
K02859	0.00000292400809476094	0.000221432691509016	0.0000488559787078402
K03390	0.00000292400809476094	0.000221432691509016	0.0000388969436833685
K03713	0.00000292400809476094	0.000221432691509016	0.000143704207602345
K03807	0.00000292400809476094	0.000221432691509016	0.0000270860008519012
K05827	0.00000292400809476094	0.000221432691509016	0.0000140197716273457
K06168	0.00000292400809476094	0.000221432691509016	0.000574167759307935
K08352	0.00000292400809476094	0.000221432691509016	0.000024028993465286
K08994	0.00000292400809476094	0.000221432691509016	0.000113220757999262
K09976	0.00000292400809476094	0.000221432691509016	0.000147116715544744
K11391	0.00000292400809476094	0.000221432691509016	0.00002085283963972
K11617	0.00000292400809476094	0.000221432691509016	0.000143375308480172
K18692	0.00000292400809476094	0.000221432691509016	0.000146102974227502
K18891	0.00000292400809476094	0.000221432691509016	0.0000949190201370871
K18892	0.00000292400809476094	0.000221432691509016	0.0000948983112071306
K19049	0.00000292400809476094	0.000221432691509016	0.000021853925658448
K00138	0.00000321343088313378	0.000221432691509016	0.0000919247955116601
K00158	0.00000321343088313378	0.000221432691509016	0.000108699817415545
K01892	0.00000321343088313378	0.000221432691509016	0.000749977430425447
K02006	0.00000321343088313378	0.000221432691509016	0.000130217065588193
K02243	0.00000321343088313378	0.000221432691509016	0.000145876168854405
K03472	0.00000321343088313378	0.000221432691509016	0.0000317800142013612
K09916	0.00000321343088313378	0.000221432691509016	0.0000313859008381164

K12308	0.00000321343088313378	0.000221432691509016	0.000374547261125537
K14982	0.00000321343088313378	0.000221432691509016	0.00011431090516968
K16869	0.00000321343088313378	0.000221432691509016	0.0000808234232535075
K17759	0.00000321343088313378	0.000221432691509016	0.000352466081458749
K18014	0.00000321343088313378	0.000221432691509016	0.0000757838530340061
K18954	0.00000321343088313378	0.000221432691509016	0.0000553322730329956
K19000	0.00000321343088313378	0.000221432691509016	0.0000224867475146454
K00452	0.0000035302207462238	0.000228338143113114	0.0000121020123445711
K01482	0.0000035302207462238	0.000228338143113114	0.0000209455030132888
K02244	0.0000035302207462238	0.000228338143113114	0.000156320856777631
K04485	0.0000035302207462238	0.000228338143113114	0.000750338651229583
K06447	0.0000035302207462238	0.000228338143113114	0.0000708742341944725
K07012	0.0000035302207462238	0.000228338143113114	0.000319549999610333
K11705	0.0000035302207462238	0.000228338143113114	0.000169820370595564
K12555	0.0000035302207462238	0.000228338143113114	0.000143621963506434
K13938	0.0000035302207462238	0.000228338143113114	0.0000307461039037346
K14983	0.0000035302207462238	0.000228338143113114	0.000134216478985991
K00839	0.00000387683493373189	0.000230104409659428	0.0000349136931858627
K01678	0.00000387683493373189	0.000230104409659428	0.000119552688881619
K01825	0.00000387683493373189	0.000230104409659428	0.0000313711025563649
K01883	0.00000387683493373189	0.000230104409659428	0.000739925987241459
K02245	0.00000387683493373189	0.000230104409659428	0.000139760919804379
K02362	0.00000387683493373189	0.000230104409659428	0.0000297775104890175
K02761	0.00000387683493373189	0.000230104409659428	0.000799576012789424
K03525	0.00000387683493373189	0.000230104409659428	0.000353255625987404

K07225	0.00000387683493373189	0.000230104409659428	0.0000253165377280844
K07570	0.00000387683493373189	0.000230104409659428	0.000147598433040097
K07637	0.00000387683493373189	0.000230104409659428	0.0000409814089119894
K09984	0.00000387683493373189	0.000230104409659428	0.0000334466329326113
K11473	0.00000387683493373189	0.000230104409659428	0.0000704515735593315
K00563	0.0000042559385263766	0.000230104409659428	0.000195169200086045
K01590	0.0000042559385263766	0.000230104409659428	0.0000118251037873293
K01868	0.0000042559385263766	0.000230104409659428	0.000740108742467166
K02240	0.0000042559385263766	0.000230104409659428	0.000147126805436559
K03664	0.0000042559385263766	0.000230104409659428	0.000726770495075672
K03693	0.0000042559385263766	0.000230104409659428	0.000143848403086787
K05340	0.0000042559385263766	0.000230104409659428	0.000154956380461052
K05812	0.0000042559385263766	0.000230104409659428	0.0000359156963784232
K06222	0.0000042559385263766	0.000230104409659428	0.0000389152432912445
K07284	0.0000042559385263766	0.000230104409659428	0.000787193089330358
K07577	0.0000042559385263766	0.000230104409659428	0.0000539912766740487
K07740	0.0000042559385263766	0.000230104409659428	0.0000302507571245606
K09945	0.0000042559385263766	0.000230104409659428	0.0000193831625631935
K11144	0.0000042559385263766	0.000230104409659428	0.00015289548992259
K14287	0.0000042559385263766	0.000230104409659428	0.000061544371058561
K17737	0.0000042559385263766	0.000230104409659428	0.0000147261729241386
K18888	0.0000042559385263766	0.000230104409659428	0.000090988227547257
K19050	0.0000042559385263766	0.000230104409659428	0.0000095925114380838
K19135	0.0000042559385263766	0.000230104409659428	0.00000210421542710367
K03278	0.00000467042080908701	0.00024137375779512	0.00000133080184589096

K05526	0.0000467042080908701	0.00024137375779512	0.0000345323114594561
K05979	0.0000467042080908701	0.00024137375779512	0.0000846532356428955
K07777	0.0000467042080908701	0.00024137375779512	0.000030090793891745
K08317	0.0000467042080908701	0.00024137375779512	0.0000791871233568476
K09024	0.0000467042080908701	0.00024137375779512	0.0000386054854378461
K09458	0.0000467042080908701	0.00024137375779512	0.000874742083693158
K09918	0.0000467042080908701	0.00024137375779512	0.0000300340568515956
K18887	0.0000467042080908701	0.00024137375779512	0.0000910361856641152
K03566	0.0000512341282561339	0.000254793119907745	0.00023786938508113
K05836	0.0000512341282561339	0.000254793119907745	0.0000911633268122536
K07251	0.0000512341282561339	0.000254793119907745	0.0000180143391291408
K09778	0.0000512341282561339	0.000254793119907745	0.0000536414495927658
K09959	0.0000512341282561339	0.000254793119907745	0.0000485352173610371
K18012	0.0000512341282561339	0.000254793119907745	0.0000698947395589224
K18013	0.0000512341282561339	0.000254793119907745	0.0000648812845512964
K19114	0.0000512341282561339	0.000254793119907745	0.0000206165416847346
K00793	0.0000561830619048016	0.0002586628915556	0.000544024528336537
K00842	0.0000561830619048016	0.0002586628915556	0.000033485549471243
K00867	0.0000561830619048016	0.0002586628915556	0.000297622963503846
K00892	0.0000561830619048016	0.0002586628915556	0.0000215710158805016
K02007	0.0000561830619048016	0.0002586628915556	0.000131508072620668
K02509	0.0000561830619048016	0.0002586628915556	0.0000441812444912146
K03046	0.0000561830619048016	0.0002586628915556	0.000748284310219433
K04064	0.0000561830619048016	0.0002586628915556	0.0000132309909810626
K05826	0.0000561830619048016	0.0002586628915556	0.0000123348327892269

K05828	0.00000561830619048016	0.0002586628915556	0.0000123664721942235
K05829	0.00000561830619048016	0.0002586628915556	0.0000123210061616346
K07075	0.00000561830619048016	0.0002586628915556	0.000236625385341313
K07497	0.00000561830619048016	0.0002586628915556	0.000865151746566149
K07652	0.00000561830619048016	0.0002586628915556	0.000155403393768606
K11358	0.00000561830619048016	0.0002586628915556	0.0000831927961125175
K15536	0.00000561830619048016	0.0002586628915556	0.0000216591858331036
K15726	0.00000561830619048016	0.0002586628915556	0.000272120079471343
K00566	0.00000615877323832001	0.000265028352047379	0.000805202678464795
K00794	0.00000615877323832001	0.000265028352047379	0.000550621694380596
K01159	0.00000615877323832001	0.000265028352047379	0.000565675691146629
K01715	0.00000615877323832001	0.000265028352047379	0.000162265385963479
K03119	0.00000615877323832001	0.000265028352047379	0.0001072157053571
K03738	0.00000615877323832001	0.000265028352047379	0.0000087007237700443
K04952	0.00000615877323832001	0.000265028352047379	0.00000547801071839679
K07082	0.00000615877323832001	0.000265028352047379	0.000722951068502611
K07498	0.00000615877323832001	0.000265028352047379	0.000514791335323759
K07709	0.00000615877323832001	0.000265028352047379	0.0000179377953769682
K08995	0.00000615877323832001	0.000265028352047379	0.0000529002997967185
K11208	0.00000615877323832001	0.000265028352047379	0.0000106769954158175
K11741	0.00000615877323832001	0.000265028352047379	0.000189808905035565
K12983	0.00000615877323832001	0.000265028352047379	0.00000584331108482591
K13626	0.00000615877323832001	0.000265028352047379	0.000101117334590232
K18824	0.00000615877323832001	0.000265028352047379	0.00000442411567964187
K01118	0.00000674878859490549	0.000279029326102308	0.000300820151128754

K02356	0.00000674878859490549	0.000279029326102308	0.000753620536916892
K03381	0.00000674878859490549	0.000279029326102308	0.0000716796241881149
K03470	0.00000674878859490549	0.000279029326102308	0.000735955927621513
K03488	0.00000674878859490549	0.000279029326102308	0.000439989769848326
K09143	0.00000674878859490549	0.000279029326102308	0.0000102397004713271
K10040	0.00000674878859490549	0.000279029326102308	0.000281918976343094
K14127	0.00000674878859490549	0.000279029326102308	0.00000971767413503127
K16850	0.00000674878859490549	0.000279029326102308	0.0000217669755046713
K19776	0.00000674878859490549	0.000279029326102308	0.00000688905021521602
K00117	0.00000739265225863569	0.000287604179936517	0.0000710305520430623
K00673	0.00000739265225863569	0.000287604179936517	0.0000871629007011518
K01338	0.00000739265225863569	0.000287604179936517	0.000656068073552144
K01923	0.00000739265225863569	0.000287604179936517	0.000741141114062021
K02996	0.00000739265225863569	0.000287604179936517	0.000731852264182725
K03330	0.00000739265225863569	0.000287604179936517	0.00000292340283777933
K03812	0.00000739265225863569	0.000287604179936517	0.0000236896417405213
K04080	0.00000739265225863569	0.000287604179936517	0.00008400772992976
K06183	0.00000739265225863569	0.000287604179936517	0.000515588804212296
K06445	0.00000739265225863569	0.000287604179936517	0.0000477843873381176
K07277	0.00000739265225863569	0.000287604179936517	0.000464461828981235
K07483	0.00000739265225863569	0.000287604179936517	0.00117728829491248
K10039	0.00000739265225863569	0.000287604179936517	0.00014106461271107
K11935	0.00000739265225863569	0.000287604179936517	0.0000149432792662228
K13479	0.00000739265225863569	0.000287604179936517	0.0000391624952788118
K16704	0.00000739265225863569	0.000287604179936517	0.00000642752987733899

K00564	0.00000809501428584255	0.00030372147905921	0.000278950296074834
K00887	0.00000809501428584255	0.00030372147905921	0.000181770280046699
K01826	0.00000809501428584255	0.00030372147905921	0.0000407231502447601
K03086	0.00000809501428584255	0.00030372147905921	0.000952123092120031
K05549	0.00000809501428584255	0.00030372147905921	0.0000293245848464961
K06981	0.00000809501428584255	0.00030372147905921	0.00000173385359305205
K08993	0.00000809501428584255	0.00030372147905921	0.0000230399952196327
K09482	0.00000809501428584255	0.00030372147905921	0.00000290692360118262
K10014	0.00000809501428584255	0.00030372147905921	0.0000279139259242503
K11535	0.00000809501428584255	0.00030372147905921	0.0000598846400703464
K02281	0.00000886090117805434	0.000312443080669655	0.00002764785522113
K02798	0.00000886090117805434	0.000312443080669655	0.000175208877933834
K02906	0.00000886090117805434	0.000312443080669655	0.000719067348104738
K03826	0.00000886090117805434	0.000312443080669655	0.000135996064628144
K07080	0.00000886090117805434	0.000312443080669655	0.000229547745296157
K07348	0.00000886090117805434	0.000312443080669655	0.00000712847177618927
K07702	0.00000886090117805434	0.000312443080669655	0.0000117034671624246
K07713	0.00000886090117805434	0.000312443080669655	0.0000729203691661377
K07803	0.00000886090117805434	0.000312443080669655	0.0000161799759901081
K09122	0.00000886090117805434	0.000312443080669655	0.0000107597583998055
K10041	0.00000886090117805434	0.000312443080669655	0.000139977091514839
K11618	0.00000886090117805434	0.000312443080669655	0.000150427346177727
K12952	0.00000886090117805434	0.000312443080669655	0.00030561044205032
K13638	0.00000886090117805434	0.000312443080669655	0.0000265925327391067
K14056	0.00000886090117805434	0.000312443080669655	0.0000133375782906298

K16511	0.00000886090117805434	0.000312443080669655	0.000163716321489529
K18581	0.00000886090117805434	0.000312443080669655	0.000159666811482601
K18765	0.00000886090117805434	0.000312443080669655	0.0000188476627054137
K01223	0.00000969574407429318	0.000326588593531224	0.00100954108253861
K01631	0.00000969574407429318	0.000326588593531224	0.0000475388884566752
K01729	0.00000969574407429318	0.000326588593531224	0.0000184143999556845
K01756	0.00000969574407429318	0.000326588593531224	0.00075345791749239
K02469	0.00000969574407429318	0.000326588593531224	0.000817990006787293
K03543	0.00000969574407429318	0.000326588593531224	0.000290909529772215
K06928	0.00000969574407429318	0.000326588593531224	0.00000946213946487025
K07013	0.00000969574407429318	0.000326588593531224	0.0000123491943233929
K07464	0.00000969574407429318	0.000326588593531224	0.000231378166999526
K09018	0.00000969574407429318	0.000326588593531224	0.0000295013678104104
K13633	0.00000969574407429318	0.000326588593531224	0.0000118985456843768
K14260	0.00000969574407429318	0.000326588593531224	0.000242077137098231
K16079	0.00000969574407429318	0.000326588593531224	0.0000664416301506332
K16849	0.00000969574407429318	0.000326588593531224	0.0000196014185990979
K00150	0.0000106054088565888	0.000331788799925862	0.0000217162476456483
K01730	0.0000106054088565888	0.000331788799925862	0.0000149626204911278
K01929	0.0000106054088565888	0.000331788799925862	0.000738261283936431
K02066	0.0000106054088565888	0.000331788799925862	0.000438238177227368
K02167	0.0000106054088565888	0.000331788799925862	0.0000263718529299944
K02340	0.0000106054088565888	0.000331788799925862	0.000715836249567066
K02345	0.0000106054088565888	0.000331788799925862	0.0000169214573671261
K02933	0.0000106054088565888	0.000331788799925862	0.000719656792412375

K03489	0.0000106054088565888	0.000331788799925862	0.0000111775407914602
K03863	0.0000106054088565888	0.000331788799925862	0.000069098014409675
K05830	0.0000106054088565888	0.000331788799925862	0.0000111645857108933
K05831	0.0000106054088565888	0.000331788799925862	0.0000112614186895884
K06191	0.0000106054088565888	0.000331788799925862	0.000217418371114052
K07104	0.0000106054088565888	0.000331788799925862	0.000176885083654664
K07639	0.0000106054088565888	0.000331788799925862	0.0000281783492488851
K07823	0.0000106054088565888	0.000331788799925862	0.000015935795742911
K08219	0.0000106054088565888	0.000331788799925862	0.00000634089504204356
K09958	0.0000106054088565888	0.000331788799925862	0.000521820277122363
K13069	0.0000106054088565888	0.000331788799925862	0.0000297022070892575
K13819	0.0000106054088565888	0.000331788799925862	0.0000341554188689528
K13933	0.0000106054088565888	0.000331788799925862	0.0000336814679026749
K14128	0.0000106054088565888	0.000331788799925862	0.0000100475017318177
K19267	0.0000106054088565888	0.000331788799925862	0.000197020939364253
K19788	0.0000106054088565888	0.000331788799925862	0.00000000141073816169145
K00441	0.0000115962282821611	0.000344391647264295	0.000012017021573745
K01244	0.0000115962282821611	0.000344391647264295	0.00000861537350084845
K01467	0.0000115962282821611	0.000344391647264295	0.000110831028637443
K01783	0.0000115962282821611	0.000344391647264295	0.000774608915890132
K02226	0.0000115962282821611	0.000344391647264295	0.000398869978836124
K02234	0.0000115962282821611	0.000344391647264295	0.0000453836994079536
K02470	0.0000115962282821611	0.000344391647264295	0.000824168098984171
K03491	0.0000115962282821611	0.000344391647264295	0.000273570626937153
K05515	0.0000115962282821611	0.000344391647264295	0.00055562371977983

K05550	0.0000115962282821611	0.000344391647264295	0.0000292785600193415
K06320	0.0000115962282821611	0.000344391647264295	0.0000324471978265274
K07303	0.0000115962282821611	0.000344391647264295	0.0000991707986035795
K07336	0.0000115962282821611	0.000344391647264295	0.0000497952601201465
K07469	0.0000115962282821611	0.000344391647264295	0.0000213089101043945
K07576	0.0000115962282821611	0.000344391647264295	0.000141449890845972
K09797	0.0000115962282821611	0.000344391647264295	0.000111452977697599
K09880	0.0000115962282821611	0.000344391647264295	0.0000213881067886734
K13016	0.0000115962282821611	0.000344391647264295	0.0000353772788853416
K00952	0.0000126689588526414	0.000363132900275518	0.00000153827610320855
K02233	0.0000126750362611582	0.000363132900275518	0.000354818599523611
K02297	0.0000126750362611582	0.000363132900275518	0.0000796108064866804
K02298	0.0000126750362611582	0.000363132900275518	0.0000795113059954364
K02931	0.0000126750362611582	0.000363132900275518	0.00072000788834864
K03328	0.0000126750362611582	0.000363132900275518	0.000264130825705993
K05359	0.0000126750362611582	0.000363132900275518	0.00000107021985813402
K09912	0.0000126750362611582	0.000363132900275518	0.0000314403481074507
K09948	0.0000126750362611582	0.000363132900275518	0.0000375527799885784
K10017	0.0000126750362611582	0.000363132900275518	0.0000244136585180085
K10775	0.0000126750362611582	0.000363132900275518	0.00000372750679556838
K11209	0.0000126750362611582	0.000363132900275518	0.000160860995637521
K18149	0.0000126750362611582	0.000363132900275518	0.0000847947285074745
K00932	0.0000138492044044299	0.000379252368924427	0.00000889499234709152
K01007	0.0000138492044044299	0.000379252368924427	0.000251464165385389
K01071	0.0000138492044044299	0.000379252368924427	0.000153444919621026

K01807	0.0000138492044044299	0.000379252368924427	0.000353993438504064
K01838	0.0000138492044044299	0.000379252368924427	0.000224056267898532
K02224	0.0000138492044044299	0.000379252368924427	0.000308172205312487
K02994	0.0000138492044044299	0.000379252368924427	0.000720527000171368
K03112	0.0000138492044044299	0.000379252368924427	0.0000262923041804544
K03919	0.0000138492044044299	0.000379252368924427	0.000033225962554988
K06603	0.0000138492044044299	0.000379252368924427	0.0000858648792964261
K07092	0.0000138492044044299	0.000379252368924427	0.00000329072965352635
K07350	0.0000138492044044299	0.000379252368924427	0.00000855467864925211
K09129	0.0000138492044044299	0.000379252368924427	0.0000236281979023169
K09920	0.0000138492044044299	0.000379252368924427	0.0000399774393033405
K11735	0.0000138492044044299	0.000379252368924427	0.0000534345626715744
K13583	0.0000138492044044299	0.000379252368924427	0.0000364707920856086
K18231	0.0000138492044044299	0.000379252368924427	0.0000543484971327194
K00123	0.0000151266809715721	0.000388030650810912	0.000387653898657604
K00840	0.0000151266809715721	0.000388030650810912	0.0000120184145330233
K01575	0.0000151266809715721	0.000388030650810912	0.000184374588637368
K01912	0.0000151266809715721	0.000388030650810912	0.000252653586557174
K02617	0.0000151266809715721	0.000388030650810912	0.0000123978396779613
K02623	0.0000151266809715721	0.000388030650810912	0.0000200428122948719
K03519	0.0000151266809715721	0.000388030650810912	0.0000720693697350265
K03569	0.0000151266809715721	0.000388030650810912	0.000681405966663967
K04069	0.0000151266809715721	0.000388030650810912	0.00081655485521012
K04078	0.0000151266809715721	0.000388030650810912	0.000748691276548971
K06208	0.0000151266809715721	0.000388030650810912	0.0000664820131041233

K07156	0.0000151266809715721	0.000388030650810912	0.0000845262770334356
K08170	0.0000151266809715721	0.000388030650810912	0.0000103562447262294
K08348	0.0000151266809715721	0.000388030650810912	0.0000152112131136482
K08484	0.0000151266809715721	0.000388030650810912	0.0000578057696616347
K10015	0.0000151266809715721	0.000388030650810912	0.0000261151480747806
K11178	0.0000151266809715721	0.000388030650810912	0.0000675693094660973
K12972	0.0000151266809715721	0.000388030650810912	0.0000805969894398574
K12973	0.0000151266809715721	0.000388030650810912	0.0000138690983744098
K13255	0.0000151266809715721	0.000388030650810912	0.0000156903463683582
K14052	0.0000151266809715721	0.000388030650810912	0.0000053224457134097
K15727	0.0000151266809715721	0.000388030650810912	0.000203808962515939
K17754	0.0000151266809715721	0.000388030650810912	0.00000255918166992185
K18456	0.0000151266809715721	0.000388030650810912	0.0000108859302545805
K19165	0.0000151266809715721	0.000388030650810912	0.000016230144846067
K19775	0.0000151266809715721	0.000388030650810912	0.00000680304357120389
K02499	0.00001651603235538	0.000407795150170425	0.00016150781774891
K04061	0.00001651603235538	0.000407795150170425	0.000117309490298274
K05790	0.00001651603235538	0.000407795150170425	0.00000583258338506558
K07110	0.00001651603235538	0.000407795150170425	0.0000764806906863028
K07460	0.00001651603235538	0.000407795150170425	0.000582210624095478
K13483	0.00001651603235538	0.000407795150170425	0.0000608236598444248
K14057	0.00001651603235538	0.000407795150170425	0.00000440070555995493
K14126	0.00001651603235538	0.000407795150170425	0.00000937125115721473
K15011	0.00001651603235538	0.000407795150170425	0.0000640598319333635
K15012	0.00001651603235538	0.000407795150170425	0.0000693286230028852

K15540	0.00001651603235538	0.000407795150170425	0.0000199718231482291
K16149	0.00001651603235538	0.000407795150170425	0.0000443203180289876
K17870	0.00001651603235538	0.000407795150170425	0.00000875453021234548
K17992	0.00001651603235538	0.000407795150170425	0.0000371355497339552
K18335	0.00001651603235538	0.000407795150170425	0.0000334272133410127
K18551	0.00001651603235538	0.000407795150170425	0.000000856733009043173
K00177	0.0000180264872449071	0.000420471803148352	0.00026172417951662
K00442	0.0000180264872449071	0.000420471803148352	0.00000335879534561359
K00691	0.0000180264872449071	0.000420471803148352	0.000152636824356979
K00927	0.0000180264872449071	0.000420471803148352	0.000738797166490075
K01453	0.0000180264872449071	0.000420471803148352	0.0000350370477514483
K02246	0.0000180264872449071	0.000420471803148352	0.000108062104888173
K02863	0.0000180264872449071	0.000420471803148352	0.000710247144803156
K03518	0.0000180264872449071	0.000420471803148352	0.00022223819881757
K05358	0.0000180264872449071	0.000420471803148352	0.0000233823610641466
K05804	0.0000180264872449071	0.000420471803148352	0.000135155516136618
K05835	0.0000180264872449071	0.000420471803148352	0.00000658892179607379
K06898	0.0000180264872449071	0.000420471803148352	0.000127998190379845
K07391	0.0000180264872449071	0.000420471803148352	0.000614311273511779
K07689	0.0000180264872449071	0.000420471803148352	0.0000254222182504167
K08350	0.0000180264872449071	0.000420471803148352	0.00000687773337385296
K09457	0.0000180264872449071	0.000420471803148352	0.000290092355019761
K09965	0.0000180264872449071	0.000420471803148352	0.0000277184696210493
K10530	0.0000180264872449071	0.000420471803148352	0.0000539939061731264
K11752	0.0000180264872449071	0.000420471803148352	0.000544794216096077

K13979	0.0000180264872449071	0.000420471803148352	0.000121774676275249
K13993	0.0000180264872449071	0.000420471803148352	0.000394834968556199
K16845	0.0000180264872449071	0.000420471803148352	0.00000409868694610295
K17329	0.0000180264872449071	0.000420471803148352	0.0000136486982179722
K18007	0.0000180264872449071	0.000420471803148352	0.0000153670278209028
K18023	0.0000180264872449071	0.000420471803148352	0.00000924223962943736
K13048	0.0000196589501226216	0.000435629309366497	0.000000117008755371978
K16956	0.0000196589501226216	0.000435629309366497	0.0000000737745477249731
K00176	0.0000196679836155224	0.000435629309366497	0.000223608416557795
K01775	0.0000196679836155224	0.000435629309366497	0.000837065443941745
K02300	0.0000196679836155224	0.000435629309366497	0.0000780026098263392
K02956	0.0000196679836155224	0.000435629309366497	0.000710133899974932
K02982	0.0000196679836155224	0.000435629309366497	0.000720126337775033
K04720	0.0000196679836155224	0.000435629309366497	0.000277599364037432
K05587	0.0000196679836155224	0.000435629309366497	0.00000396115886357909
K06876	0.0000196679836155224	0.000435629309366497	0.000064363357227882
K07458	0.0000196679836155224	0.000435629309366497	0.000128867375263827
K07644	0.0000196679836155224	0.000435629309366497	0.0000795185414890839
K07739	0.0000196679836155224	0.000435629309366497	0.00000842862897279458
K10748	0.0000196679836155224	0.000435629309366497	0.0000162411940344081
K11041	0.0000196679836155224	0.000435629309366497	0.000100454446813508
K11811	0.0000196679836155224	0.000435629309366497	0.0000311217429191637
K13060	0.0000196679836155224	0.000435629309366497	0.0000286657199208909
K15532	0.0000196679836155224	0.000435629309366497	0.000172093896620253
K15780	0.0000196679836155224	0.000435629309366497	0.0000779018929100923

K16846	0.0000196679836155224	0.000435629309366497	0.00000367677986059957
K17240	0.0000196679836155224	0.000435629309366497	0.00000741222339700479
K18299	0.0000196679836155224	0.000435629309366497	0.00000921142364555291
K18932	0.0000196679836155224	0.000435629309366497	0.000000000384988425045645
K19714	0.0000196679836155224	0.000435629309366497	0.0000078206710054425
K19737	0.0000214414515912223	0.000446956914548294	0.000000000637949509570311
K00174	0.0000214512187006959	0.000446956914548294	0.000446048194045338
K00215	0.0000214512187006959	0.000446956914548294	0.000714135206113125
K00500	0.0000214512187006959	0.000446956914548294	0.0000672973770802796
K00627	0.0000214512187006959	0.000446956914548294	0.000518089766260736
K00694	0.0000214512187006959	0.000446956914548294	0.0000984811172832143
K00891	0.0000214512187006959	0.000446956914548294	0.000704633038525815
K01358	0.0000214512187006959	0.000446956914548294	0.000907768963529624
K01829	0.0000214512187006959	0.000446956914548294	0.0000286973914765697
K01950	0.0000214512187006959	0.000446956914548294	0.000356264566422878
K02225	0.0000214512187006959	0.000446956914548294	0.0000474246778649206
K02385	0.0000214512187006959	0.000446956914548294	0.0000896946329119102
K02600	0.0000214512187006959	0.000446956914548294	0.000749668799301328
K03647	0.0000214512187006959	0.000446956914548294	0.000236433825338674
K05800	0.0000214512187006959	0.000446956914548294	0.000119171252084568
K05919	0.0000214512187006959	0.000446956914548294	0.000186100237417565
K06937	0.0000214512187006959	0.000446956914548294	0.0000326594124072282
K07065	0.0000214512187006959	0.000446956914548294	0.0000184682464292047
K07217	0.0000214512187006959	0.000446956914548294	0.00008970838127682
K07233	0.0000214512187006959	0.000446956914548294	0.0000519846909634965

K07477	0.0000214512187006959	0.000446956914548294	0.0000502905275253772
K07506	0.0000214512187006959	0.000446956914548294	0.0000967298625243613
K07700	0.0000214512187006959	0.000446956914548294	0.000120648699085585
K09951	0.0000214512187006959	0.000446956914548294	0.000532204794476112

Table S1D. Relative abundance of the most significant KEGG metabolic pathways in the samples collected in periodontitis sites and healthy sites as assessed by Mann-Whitney U tests with false-discovery-rate correction.

**Feature**	**Feature**	**P value**	**FDR corrected P value**	**mean relative abundance healthy sites**	**mean relative abudance diseased sites**
reductive acetyl coenzyme A pathway	CODH-PWY	0.000000221219627049992	0.0000657368645675495	0.000494926518447968	0.00135069367215016
1,4-dihydroxy-6-naphthoate biosynthesis I	PWY-7374	0.000000301747276184077	0.0000657368645675495	0.000134879291272032	0.000400221748031889
superpathway of menaquinol-8 biosynthesis II	PWY-6263	0.000000502546242123424	0.0000657368645675495	0.000674538621947707	0.00166974511122918
1,4-dihydroxy-6-naphthoate biosynthesis II	PWY-7371	0.00000055591428809767	0.0000657368645675495	0.000291510171363232	0.000796956753344786
peptidoglycan biosynthesis IV (Enterococcus faecium)	PWY-6471	0.0000035302207462238	0.000203749213739465	0.00353555012034855	0.00206284767768124
L-arginine degradation II (AST pathway)	AST-PWY	0.00000387683493373189	0.000203749213739465	0.000368631543101274	0.00004765354045556
NAD biosynthesis II (from tryptophan)	NADSYN-PWY	0.00000387683493373189	0.000203749213739465	0.000274024987771599	0.0000548057368216869

incomplete reductive TCA cycle	P42-PWY	0.00000387683493373189	0.000203749213739465	0.00533160345230498	0.00652419241665213
L-tryptophan degradation to 2-amino-3-carboxymuconate semialdehyde	PWY-5651	0.00000387683493373189	0.000203749213739465	0.000181033735836268	0.0000345184176606504
superpathway of demethylmenaquinol-6 biosynthesis II	PWY-7373	0.00000512341282561339	0.000242337426651513	0.000161722777380462	0.000472951615195553
peptidoglycan biosynthesis V (β-lactam resistance)	PWY-6470	0.00000674878859490549	0.000268978809102668	0.00218147952169114	0.00102997421055483
L-methionine salvage cycle I (bacteria and plants)	PWY-7528	0.00000739265225863569	0.000268978809102668	6.94805810427789E-06	0.0000339470415901102
superpathway of thiamin diphosphate biosynthesis I	THISYN-PWY	0.00000739265225863569	0.000268978809102668	0.00364290145180392	0.00467817527700594
L-lysine fermentation to acetate and butanoate	P163-PWY	0.00000809501428584255	0.000273495839800252	0.00111628424736602	0.0026071490072827
pyrimidine deoxyribonucleotides de novo biosynthesis III	PWY-6545	0.00000969574407429318	0.00028868505144538	0.00239603429787342	0.00345152914188192
L-glutamate degradation V (via hydroxyglutarate)	P162-PWY	0.0000106054088565888	0.00028868505144538	0.00118412883284125	0.00218286084440245
thiazole biosynthesis I (E. coli)	PWY-6892	0.0000106054088565888	0.00028868505144538	0.00276141451662597	0.00417289160962233
enterobacterial common antigen biosynthesis	ECASYN-PWY	0.0000115962282821611	0.00028868505144538	0.000108454862303882	0.0000227667533238225
superpathway of L-alanine biosynthesis	PWY0-1061	0.0000115962282821611	0.00028868505144538	0.00389405923344301	0.00183838541754824

hexitol fermentation to lactate, formate, ethanol and acetate	P461-PWY	0.0000180264872449071	0.000387569475765502	0.00208535479303114	0.0009435725170468
pyrimidine deoxyribonucleotides de novo biosynthesis IV	PWY-7198	0.0000180264872449071	0.000387569475765502	0.000260986553566879	0.000623906169274919
pyrimidine deoxyribonucleotides biosynthesis from CTP	PWY-7210	0.0000180264872449071	0.000387569475765502	0.000362884524019541	0.000824782073742826
chorismate biosynthesis II (archaea)	PWY-6165	0.0000196589501226216	0.000387623177089254	0.0000105557876060832	0.000052968195195632
acetylene degradation	P161-PWY	0.0000196679836155224	0.000387623177089254	0.0037116469781165	0.00200271812489983
pyruvate fermentation to acetone	PWY-6588	0.0000214512187006959	0.000390247170978044	0.00250570601796752	0.004521814493671
superpathway of thiamin diphosphate biosynthesis II	PWY-6895	0.0000214512187006959	0.000390247170978044	0.00297605326821761	0.0041767805925807
flavin biosynthesis I (bacteria and plants)	RIBOSYN2-PWY	0.0000233877021067009	0.000409717892461834	0.00523853558421813	0.0063263527262042
adenosylcobalamin salvage from cobinamide I	COBALSYN-PWY	0.0000277708562078396	0.000444998440217873	0.00325134738610232	0.00513387613232505
superpathway of demethylmenaquinol-6 biosynthesis I	PWY-5860	0.0000302451334151159	0.000444998440217873	0.00088622184668419	0.000392196498902103
adenosylcobalamin biosynthesis from cobyrinate a,c-diamide I	PWY-5509	0.0000329280029759525	0.000444998440217873	0.0031599678348824	0.0049989066675955
5-aminoimidazole ribonucleotide biosynthesis II	PWY-6122	0.0000329280029759525	0.000444998440217873	0.00682146433406757	0.00764241789623689

CMP-pseudaminate biosynthesis	PWY-6143	0.0000329280029759525	0.000444998440217873	0.0000981167999230143	0.000349141463134474
superpathway of salicylate degradation	PWY-6182	0.0000329280029759525	0.000444998440217873	0.000345040490450165	0.0000631047546886317
adenosylcobalamin salvage from cobinamide II	PWY-6269	0.0000329280029759525	0.000444998440217873	0.00318145870260126	0.0050429912498333
superpathway of 5-aminoimidazole ribonucleotide biosynthesis	PWY-6277	0.0000329280029759525	0.000444998440217873	0.00682146433406757	0.00764241789623689
superpathway of menaquinol-6 biosynthesis I	PWY-5850	0.0000358359552047385	0.000446063337153719	0.00114429116985453	0.000554685863628255
superpathway of menaquinol-10 biosynthesis	PWY-5896	0.0000358359552047385	0.000446063337153719	0.00114429116985453	0.000554685863628255
inosine-5'-phosphate biosynthesis III	PWY-7234	0.0000358359552047385	0.000446063337153719	0.00424215510939054	0.00264892882501515
ADP-L-glycero-β-D-manno-heptose biosynthesis	PWY0-1241	0.0000460938008457867	0.00051508010256418	0.00134109739810731	0.0020792352770563
1,3-propanediol biosynthesis (engineered)	PWY-7385	0.0000500714942768574	0.00051508010256418	2.76734628774204E-06	0.0000101611768610968
Calvin-Benson-Bassham cycle	CALVIN-PWY	0.0000500923566975736	0.00051508010256418	0.000452735549021858	0.0000926603483634548
catechol degradation III (ortho-cleavage pathway)	PWY-5417	0.0000500923566975736	0.00051508010256418	0.000452735549021858	0.0000926603483634548
aromatic compounds degradation via β-ketoadipate	PWY-5431	0.0000500923566975736	0.00051508010256418	0.00088622184668419	0.000417487467232591

superpathway of demethylmenaquinol-9 biosynthesis	PWY-5862	0.0000500923566975736	0.00051508010256418	0.0000217186537118378	0.000119794532606461
mevalonate pathway II (archaea)	PWY-6174	0.0000500923566975736	0.00051508010256418	0.00342380385495024	0.00235705063339875
superpathway of purine deoxyribonucleosides degradation	PWY0-1297	0.000054418226124023	0.000527407858959796	0.0013045357393493	0.000648967651360971
superpathway of chorismate metabolism	ALL-CHORISMATE-PWY	0.000054418226124023	0.000527407858959796	0.00679522770743061	0.00750457691383069
5-aminoimidazole ribonucleotide biosynthesis I	PWY-6121	0.0000590722747866476	0.000527407858959796	4.44347617111497E-06	0.0000185410491179205
L-lysine biosynthesis V	PWY-3081	0.0000590964408559603	0.000527407858959796	0.00485792086460714	0.00545220622299064
pantothenate and coenzyme A biosynthesis I	PANTOSYN-PWY	0.0000590964408559603	0.000527407858959796	0.00114429116985453	0.000590964833435688
superpathway of menaquinol-9 biosynthesis	PWY-5845	0.0000590964408559603	0.000527407858959796	0.00734364753085983	0.00840969238812507
adenosine ribonucleotides de novo biosynthesis	PWY-7219	0.0000590964408559603	0.000527407858959796	0.00347583622283514	0.00463096420804477
superpathway of GDP-mannose-derived O-antigen building blocks biosynthesis	PWY-7323	0.0000641537954385207	0.000551722640771278	0.0003021618050931	0.0000634617195956713
gallate degradation I	GALLATE-DEGRADATION-II-PWY	0.0000641537954385207	0.000551722640771278	0.00653582754535598	0.00714709663310739
UMP biosynthesis	PWY-5686	0.0000696189589734684	0.000588031564186617	0.0026089517161654	0.00134932706735347

heterolactic fermentation	P122-PWY	0.0000755225929815156	0.000605460787800964	0.00121729616447853	0.00172401377754231
superpathway of polyamine biosynthesis I	POLYAMSYN-PWY	0.0000755225929815156	0.000605460787800964	0.00216442044848664	0.0010545454258859
sucrose degradation IV (sucrose phosphorylase)	PWY-5384	0.0000755225929815156	0.000605460787800964	0.00627462517534912	0.00703977870125012
chorismate biosynthesis from 3-dehydroquinate	PWY-6163	0.0000818974756486839	0.000635041081669303	0.00108902399817297	0.0021416630209193
succinate fermentation to butanoate	PWY-5677	0.0000818974756486839	0.000635041081669303	0.00298950723942221	0.00395261248048411
preQ0 biosynthesis	PWY-6703	0.0000887786327270478	0.000666544337776089	0.00165925497982006	0.00275193710449413
thiazole biosynthesis II (Bacillus)	PWY-6891	0.0000887786327270478	0.000666544337776089	0.00161231333297203	0.00253768292109067
cob(II)yrinate a,c-diamide biosynthesis I (early cobalt insertion)	PWY-7377	0.000096203475366264	0.000711003810128795	0.00429465711787303	0.00287842480734086
peptidoglycan maturation (meso-diaminopimelate containing)	PWY0-1586	0.000104211945158089	0.000724886030290826	0.0027938943549531	0.00190805543199263
superpathway of hexitol degradation (bacteria)	HEXITOLDEGSUPER-PWY	0.000104211945158089	0.000724886030290826	0.00292984134042714	0.00149821072686197
TCA cycle IV (2-oxoglutarate decarboxylase)	P105-PWY	0.000104211945158089	0.000724886030290826	0.00136924638065944	0.000602116711329558
superpathway of heme biosynthesis from glycine	PWY-5920	0.000104211945158089	0.000724886030290826	0.00022613307815331	0.0000480781930607917

4-methylcatechol degradation (ortho cleavage)	PWY-6185	0.000112803849541105	0.000762521047719722	3.04366974181011E-08	2.80787774947345E-07
L-selenocysteine biosynthesis II (archaea and eukaryotes)	PWY-6281	0.000112846666681566	0.000762521047719722	0.00404936219899596	0.00316870623981894
mixed acid fermentation	FERMENTATION-PWY	0.000122107200185148	0.000780789459582707	1.45616335305813E-06	5.30836934777156E-06
3-hydroxypropanoate cycle	PWY-5743	0.000122107200185148	0.000780789459582707	1.19564269925353E-08	3.25897680512628E-07
superpathway of methanogenesis	PWY-6830	0.000122153107841692	0.000780789459582707	0.00514910785306992	0.00637726192797503
superpathway of geranylgeranyl diphosphate biosynthesis II (via MEP)	PWY-5121	0.000122153107841692	0.000780789459582707	0.00495312891630358	0.00432935162134697
pyrimidine deoxyribonucleotides de novo biosynthesis I	PWY-7184	0.000132130546837205	0.000791405328423846	1.20480619307481E-07	6.50001136954347E-07
3-hydroxypropanoate/4-hydroxybutanate cycle	PWY-5789	0.00013217974829912	0.000791405328423846	0.000201881166475196	0.0000471854800363959
4-hydroxyphenylacetate degradation	3-HYDROXYPHENYLACETATE-DEGRADATION-PWY	0.00013217974829912	0.000791405328423846	0.00291624046344008	0.00165336192453742
Bifidobacterium shunt	P124-PWY	0.00013217974829912	0.000791405328423846	0.000309962543127924	0.0000706363191155441
toluene degradation III (aerobic) (via p-cresol)	PWY-5181	0.00013217974829912	0.000791405328423846	0.000758744201622007	0.00032878179623362

L-tyrosine degradation I	TYRFUMCAT-PWY	0.000142925545906715	0.000834922410203152	1.21151273398591E-07	7.02005064091424E-07
6-hydroxymethyl-dihydropterin diphosphate biosynthesis II (archaea)	PWY-6797	0.000142978256292717	0.000834922410203152	0.00620590741372807	0.0071402268008011
inosine-5'-phosphate biosynthesis I	PWY-6123	0.000154603674160626	0.000881054673228626	0.00140602280668983	0.000927340729964588
urea cycle	PWY-4984	0.000154603674160626	0.000881054673228626	0.000192903431835602	0.0000518021680809586
superpathway of L-threonine metabolism	THREOCAT-PWY	0.000167114612868712	0.000929943669257658	0.00530024737334159	0.00681975556948946
methylerythritol phosphate pathway I	NONMEVIPP-PWY	0.000167114612868712	0.000929943669257658	0.00530024737334159	0.00681975556948946
methylerythritol phosphate pathway II	PWY-7560	0.000180508800957256	0.000981738443917436	2.68220849050142E-08	9.21655931886056E-08
crotonate fermentation (to acetate and cyclohexane carboxylate)	PWY-7401	0.000180573455857964	0.000981738443917436	0.00180823469535415	0.000704707162546314
superpathway of glyoxylate bypass and TCA	TCA-GLYOX-BYPASS	0.000194977418142969	0.000995587853151743	1.89432139758495E-06	6.20941805230835E-06
starch biosynthesis	PWY-622	0.000194977418142969	0.000995587853151743	2.30508564704392E-09	7.9216332100888E-09
superpathway of seleno-compound metabolism	PWY-6395	0.000195046572524399	0.000995587853151743	0.000455240403643121	0.000933076537645966
UDP-2,3-diacetamido-2,3-dideoxy-α-D-mannuronate biosynthesis	PWY-7090	0.000210530604502751	0.000995587853151743	2.60144256214352E-09	8.97035058734885E-09

paromamine biosynthesis II	PWY-7022	0.000210604541646389	0.000995587853151743	0.000306421898856591	0.0000677728361329049
gallate degradation II	GALLATE-DEGRADATION-I-PWY	0.000210604541646389	0.000995587853151743	0.00037327696493533	0.0000844890056735354
methylgallate degradation	METHYLGALLATE-DEGRADATION-PWY	0.000210604541646389	0.000995587853151743	0.00335394733603082	0.00503691326512718
pyruvate fermentation to propanoate I	P108-PWY	0.000210604541646389	0.000995587853151743	6.03840578967739E-06	0.000019844304800106
Entner-Doudoroff pathway III (semi-phosphorylative)	PWY-2221	0.000210604541646389	0.000995587853151743	0.000134159803271314	0.0000402206457637073
L-tryptophan degradation IX	PWY-5655	0.000210604541646389	0.000995587853151743	2.09422088531732E-06	7.00047819959841E-06
nitrifier denitrification	PWY-7084	0.000227243366311467	0.000995587853151743	1.74636271801509E-07	5.76644842922751E-07
L-isoleucine degradation I	ILEUDEG-PWY	0.000227243366311467	0.000995587853151743	2.0850185050627E-08	6.94046875171908E-08
Entner-Doudoroff pathway II (non-phosphorylative)	NPGLUCAT-PWY	0.000227243366311467	0.000995587853151743	1.72851935930711E-07	5.70728827104619E-07
folate transformations I	PWY-2201	0.000227243366311467	0.000995587853151743	4.67378867689059E-07	1.54328170558121E-06
nitrate reduction V (assimilatory)	PWY-5675	0.000227243366311467	0.000995587853151743	1.20570185027976E-06	4.00490875696286E-06
glyoxylate assimilation	PWY-5744	0.000227243366311467	0.000995587853151743	1.73388722699059E-06	5.70533367140109E-06

superpathway of the 3-hydroxypropanoate cycle	PWY-7024	0.000227322385074817	0.000995587853151743	0.0004748473312594	0.000107777258035632
catechol degradation to β-ketoadipate	CATECHOL-ORTHO-CLEAVAGE-PWY	0.000227322385074817	0.000995587853151743	0.00845294111715122	0.00936762559675867
pentose phosphate pathway (non-oxidative branch)	NONOXIPENT-PWY	0.000227322385074817	0.000995587853151743	0.00473422770203015	0.00387322091399136
pentose phosphate pathway	PENTOSE-P-PWY	0.000227322385074817	0.000995587853151743	0.00239600482581123	0.00385656303605431
L-glutamate and L-glutamine biosynthesis	PWY-5505	0.000227322385074817	0.000995587853151743	0.000053044200867461	0.000137298737668777
ergothioneine biosynthesis I (bacteria)	PWY-7255	0.000236853732198821	0.00102781481954167	9.9692796286453E-09	4.51017892271445E-08
superpathway of sialic acids and CMP-sialic acids biosynthesis	PWY-6145	0.000244942276996176	0.00103586920604104	1.15410793860225E-09	3.8205156369048E-09
streptomycin biosynthesis	PWY-5940	0.00024527981200126	0.00103586920604104	0.00358211860911261	0.00445113304072093
colanic acid building blocks biosynthesis	COLANSYN-PWY	0.00024527981200126	0.00103586920604104	0.00243337685981569	0.0012539578387922
superpathway of glycolysis, pyruvate dehydrogenase, TCA, and glyoxylate bypass	GLYCOLYSIS-TCA-GLYOX-BYPASS	0.000264201030447092	0.00107877221774065	1.50446121613556E-09	4.97239979173245E-09
neopentalenoketolactone and pentalenate biosynthesis	PWY-6919	0.000264561474118216	0.00107877221774065	0.00181416822018896	0.000656436963263688
fatty acid β-oxidation I	FAO-PWY	0.000264561474118216	0.00107877221774065	0.00493888919069376	0.00319605215725218

sucrose degradation III (sucrose invertase)	PWY-621	0.000264561474118216	0.00107877221774065	0.00025056771194711	0.0000780540881985392
phenylacetate degradation I (aerobic)	PWY0-321	0.000284296057683284	0.00112438892273416	3.30367704639228E-10	9.76059061916559E-10
ribostamycin biosynthesis	PWY-7015	0.000285257231983296	0.00112438892273416	0.000122397462020045	0.0000308980027527626
superpathway of L-arginine, putrescine, and 4-aminobutanoate degradation	ARGDEG-PWY	0.000285257231983296	0.00112438892273416	0.00336223422829096	0.00454963093676035
lipid IVA biosynthesis	NAGLIPASYN-PWY	0.000285257231983296	0.00112438892273416	0.000122397462020045	0.0000308980027527626
superpathway of L-arginine and L-ornithine degradation	ORNARGDEG-PWY	0.0003070519008775 7	0.00116343784607939	6.49140647224054E-10	2.13605215297629E-09
paromamine biosynthesis I	PWY-7014	0.0003070519008775 7	0.00116343784607939	6.78411456715374E-10	2.23725197709979E-09
mevalonate pathway III (archaea)	PWY-7524	0.000307462432896244	0.00116343784607939	0.00613623728529322	0.0065091636512551
chorismate biosynthesis I	ARO-PWY	0.000307462432896244	0.00116343784607939	0.00603464791906958	0.00670342015240473
coenzyme A biosynthesis I	COA-PWY	0.000307462432896244	0.00116343784607939	0.00569909982338972	0.00514927408154113
superpathway of guanosine nucleotides de novo biosynthesis I	PWY-7228	0.000331278200593418	0.00124360784825942	0.000236153759226996	0.000113510296564178
superpathway of glycerol degradation to 1,3-propanediol	GOLPDLCAT-PWY	0.000356811737059108	0.00132891300495243	0.00638338436940538	0.00729395393587246

tRNA charging	TRNA-CHARGING-PWY	0.000384052219405384	0.00141919296702146	0.0000174945394903833	0.0000607909459753789
reductive TCA cycle II	PWY-5392	0.000413360675461939	0.00151565581002711	1.98306250228997E-06	4.98606794753847E-06
sitosterol degradation to androstenedione	PWY-6948	0.000444888840408926	0.00161871093471863	0.0073809787628163	0.00822465974745094
superpathway of adenosine nucleotides de novo biosynthesis I	PWY-7229	0.000477897619295292	0.00168902759191516	4.9196137027867E-10	1.45076198432908E-09
superpathway of butirocin biosynthesis	PWY-7020	0.000478348082313356	0.00168902759191516	0.0000229259970332246	0.000116587897573188
coenzyme B biosynthesis	P241-PWY	0.000478498302994993	0.00168902759191516	0.00702593430708791	0.00786430767457763
superpathway of adenosine nucleotides de novo biosynthesis II	PWY-6126	0.000478498302994993	0.00168902759191516	0.000104846795875007	0.000275275512326133
protein N-glycosylation (bacterial)	PWY-7031	0.000514464165759173	0.00180253000299325	0.0034740253062232	0.00435509194295818
CMP-3-deoxy-D-manno-octulosonate biosynthesis I	PWY-1269	0.000552937152555178	0.00189521212433767	0.000185460208450963	0.0000598853515981832
toluene degradation IV (aerobic) (via catechol)	PWY-5178	0.000552937152555178	0.00189521212433767	0.00626508055770858	0.00684201574101312
peptidoglycan biosynthesis III (mycobacteria)	PWY-6385	0.000552937152555178	0.00189521212433767	0.00129779371021505	0.000603241493661769
mevalonate pathway I	PWY-922	0.000594076540877892	0.00197886059038904	1.99956857664599E-06	6.11725390593094E-06

benzoyl-CoA degradation II (anaerobic)	CENTBENZCOA-PWY	0.000594076540877892	0.00197886059038904	0.0000413594106049813	0.000173335810038483
coenzyme M biosynthesis I	P261-PWY	0.000594076540877892	0.00197886059038904	0.0000636654468686651	0.000180301416251358
sucrose degradation II (sucrose synthase)	PWY-3801	0.000594076540877892	0.00197886059038904	0.000173821777935861	0.0000289814995253523
polymyxin resistance	PWY0-1338	0.00063805057031995 9	0.00203917513352257	0.000138715181270575	0.000272273754638336
glycolysis V (Pyrococcus)	P341-PWY	0.000638050570319959	0.00203917513352257	0.00201182652566081	0.00293323236755288
acetyl-CoA fermentation to butanoate II	PWY-5676	0.000638050570319959	0.00203917513352257	0.0016238293377541	0.000814694516367718
superpathway of geranylgeranyldiphosphate biosynthesis I (via mevalonate)	PWY-5910	0.000638050570319959	0.00203917513352257	0.000915605345681968	0.00050004473913492
superpathway of 2,3-butanediol biosynthesis	PWY-6396	0.000638050570319959	0.00203917513352257	0.000450023320672417	0.000148688406627122
2-methylcitrate cycle I	PWY0-42	0.000638050570319959	0.00203917513352257	0.00327874730027366	0.00235999821356919
TCA cycle VIII (helicobacter)	REDCITCYC	0.000684832877573556	0.00210404180080762	1.19688490564676E-06	0.000041279683258374
CDP-archaeol biosynthesis	PWY-6349	0.000684832877573556	0.00210404180080762	1.41134924073927E-06	0.0000422811063142697
archaetidylinositol biosynthesis	PWY-6350	0.000685036865379226	0.00210404180080762	0.00592218075763872	0.00635980263495448

gluconeogenesis I	GLUCONEO-PWY	0.000685036865379226	0.00210404180080762	0.00633523586955355	0.0069037710470221
peptidoglycan biosynthesis I (meso-diaminopimelate containing)	PEPTIDOGLYCANSYN-PWY	0.000685036865379226	0.00210404180080762	0.00049073141124694	0.000109406480177776
syringate degradation	PWY-6339	0.000685036865379226	0.00210404180080762	0.000461391772241657	0.00079087858873943
methylaspartate cycle	PWY-6728	0.000735222872785861	0.00224361560534008	0.00630811349924582	0.00667094524936986
superpathway of aromatic amino acid biosynthesis	COMPLETE-ARO-PWY	0.000788576428136376	0.00234657475649965	0.0000157275538202767	0.000110121816706357
methanogenesis from H2 and CO2	METHANOGENESIS-PWY	0.000788806313495655	0.00234657475649965	0.00179827330776377	0.0022502900332244
superpathway of arginine and polyamine biosynthesis	ARG+POLYAMINE-SYN	0.000788806313495655	0.00234657475649965	0.00401825725543351	0.00332215034941289
L-methionine biosynthesis III	HSERMETANA-PWY	0.000788806313495655	0.00234657475649965	0.000101583735116364	0.0000386630767688974
superpathway of aerobic toluene degradation	PWY-5183	0.000843558871246708	0.00245494443067596	4.46387012303079E-10	1.22426394584108E-09
butirosin biosynthesis	PWY-7019	0.000845995649471841	0.00245494443067596	0.000780594205900617	0.000306705620093583
protocatechuate degradation II (ortho-cleavage pathway)	PROTOCATECHUATE-ORTHO-CLEAVAGE-PWY	0.000845995649471841	0.00245494443067596	0.000193496035140351	0.0000674577271006742
superpathway of phenylethylamine degradation	PWY-6071	0.000845995649471841	0.00245494443067596	0.00160129142205948	0.000740415498396242

fatty acid salvage	PWY-7094	0.000906751918997468	0.00256895806833111	1.27331214377164E-06	0.0000552000519532944
tetrahydromethanopterin biosynthesis	PWY-6148	0.000907010565351574	0.00256895806833111	0.00460939418636855	0.00390469560322484
superpathway of histidine, purine, and pyrimidine biosynthesis	PRPP-PWY	0.000907010565351574	0.00256895806833111	0.0000860713099602577	0.0000256771659554684
2-nitrobenzoate degradation I	PWY-5647	0.000907010565351574	0.00256895806833111	0.00433745161985592	0.00355484532325813
superpathway of pyrimidine deoxyribonucleotides de novo biosynthesis	PWY-7211	0.000926274873881101	0.00259247346358438	2.260001962782E-11	9.96325569585817E-11
superpathway of erythromycin biosynthesis	PWY-6977	0.000926274873881101	0.00259247346358438	2.260001962782E-11	9.68681852649075E-11
superpathway of megalomicin A biosynthesis	PWY-7110	0.000971808284644813	0.00268885968406864	3.39140096368807E-07	8.50700969741689E-07
cholesterol degradation to androstenedione II (cholesterol dehydrogenase)	PWY-6946	0.000972082465064983	0.00268885968406864	0.00437408769977867	0.00390977967959306
pyrimidine deoxyribonucleotide phosphorylation	PWY-7197	0.0010414549834444	0.00284744628421504	0.00146374467517256	0.000669333821834835
glyoxylate cycle	GLYOXYLATE-BYPASS	0.0010414549834444	0.00284744628421504	0.00644089868622417	0.00699848707662669
UDP-N-acetylmuramoyl-pentapeptide biosynthesis I (meso-diaminopimelate containing)	PWY-6387	0.00111507678921774	0.00299759587822772	1.15876115842117E-06	0.0000594925355406848
phosphopantothenate biosynthesis III	PWY-6654	0.00111538451282892	0.00299759587822772	0.000242177654701391	0.000433537656095744

methanogenesis from acetate	METH-ACETATE-PWY	0.00111538451282892	0.00299759587822772	0.00327238431058866	0.00253171112882813
superpathway of pyrimidine deoxyribonucleosides degradation	PWY0-1298	0.00113321924239812	0.00302832034832945	6.36551430173179E-12	4.61114511692195E-11
erythromycin D biosynthesis	PWY-7106	0.0011941407446348	0.00313793651229033	0.00116205917952899	0.000579965596822105
lactose and galactose degradation I	LACTOSECAT-PWY	0.0011941407446348	0.00313793651229033	1.19027733081359E-06	2.74649239934819E-06
toluene degradation VI (anaerobic)	PWY-5184	0.0011941407446348	0.00313793651229033	0.000581408764737014	0.000189062833531447
2-methylcitrate cycle II	PWY-5747	0.00127662838454068	0.00321541179689053	1.32573724435626E-06	1.36789989450808E-09
superpathway of mycolyl-arabinogalactan-peptidoglycan complex biosynthesis	PWY-6404	0.0012776624239361	0.00321541179689053	0.0000369102428316905	0.000118421336375411
gluconeogenesis II (Methanobacterium thermoautotrophicum)	PWY-6142	0.0012776624239361	0.00321541179689053	1.03307648511157E-06	0.0000932126680374041
flavin biosynthesis II (archaea)	PWY-6167	0.0012776624239361	0.00321541179689053	3.49758151579925E-07	0.0000399664216494431
7-(3-amino-3-carboxypropyl)-wyosine biosynthesis	PWY-7286	0.00127800722582541	0.00321541179689053	0.00665733054128903	0.00712833803802195
glycolysis I (from glucose 6-phosphate)	GLYCOLYSIS	0.00127800722582541	0.00321541179689053	0.00633469928953158	0.00688286915884499
UDP-N-acetylmuramoyl-pentapeptide biosynthesis II (lysine-containing)	PWY-6386	0.00127800722582541	0.00321541179689053	0.000581482203621785	0.00102180521335913

GDP-D-glycero-α-D-manno-heptose biosynthesis	PWY-6478	0.00127800722582541	0.00321541179689053	0.00153563838531518	0.000983332468582862
superpathway of heme biosynthesis from uroporphyrinogen-III	PWY0-1415	0.00136728193017532	0.00333363068542745	0.0035334902442853	0.00290532164519893
L-ornithine biosynthesis	GLUTORN-PWY	0.00136728193017532	0.00333363068542745	0.00130089909297954	0.00194529354141476
photorespiration	PWY-181	0.00136728193017532	0.00333363068542745	0.00589100702966312	0.00671175414565785
pyruvate fermentation to acetate and lactate II	PWY-5100	0.00136728193017532	0.00333363068542745	0.00667962545643952	0.00711781505738364
guanosine ribonucleotides de novo biosynthesis	PWY-7221	0.00136728193017532	0.00333363068542745	0.00342344109232544	0.00250803191972699
purine ribonucleosides degradation	PWY0-1296	0.00136728193017532	0.00333363068542745	0.000504697760101567	0.000243729282167928
methylphosphonate degradation I	PWY0-1533	0.00146189213374085	0.00352886438927517	0.0000075014365694816	0.000019783689572111
mannosylglycerate biosynthesis I	PWY-5656	0.00146227784418168	0.00352886438927517	0.00720551867644522	0.00792020724971901
superpathway of pyrimidine nucleobases salvage	PWY-7208	0.00156332356743264	0.00375356369236364	0.000211037286996612	0.0000631124995530554
3-phenylpropanoate degradation	P281-PWY	0.00178450567006235	0.00426298576737118	0.0000013343687202148	0.0000470988759999314
Methanobacterium thermoautotrophicum biosynthetic metabolism	PWY-6146	0.00203465099939274	0.00481314821421337	3.13100494043165E-07	0.0000362517598462128

archaetidylserine and archaetidylethanolamine biosynthesis	PWY-6141	0.00203515780727838	0.00481314821421337	0.0008446619190102	0.000458072265419947
myo-inositol degradation I	P562-PWY	0.0021719718205802 3	0.00508585480759628	0.00268227727602228	0.00365384990690034
L-histidine degradation I	HISDEG-PWY	0.00217197182058023	0.00508585480759628	0.0000182514404655821	0.0000483098247217487
androstenedione degradation	PWY-6944	0.00228012317408728	0.00526933481116585	2.51504915343216E-12	6.65411457118316E-11
pentalenolactone biosynthesis	PWY-6915	0.0022809798815085	0.00526933481116585	6.36551430173179E-12	4.36311252483613E-11
tylosin biosynthesis	PWY-7415	0.0023171704877854	0.00526933481116585	0.000259634405577346	0.0000765524884887063
superpathway of ornithine degradation	ORNDEG-PWY	0.0023171704877854	0.00526933481116585	0.00415774703984664	0.00378884920970159
superpathway of pyrimidine deoxyribonucleoside salvage	PWY-7200	0.0023171704877854	0.00526933481116585	0.00327278856313204	0.00218339170019251
D-fructuronate degradation	PWY-7242	0.0023171704877854	0.00526933481116585	0.00420927659792734	0.00363410421680313
anhydromuropeptides recycling	PWY0-1261	0.00247061560840068	0.00553972592594905	5.07689144000859E-07	1.45293313814208E-06
xylose degradation III	PWY-6760	0.00247120966252696	0.00553972592594905	0.00127996053571664	0.000570395524344194
enterobactin biosynthesis	ENTBACSYN-PWY	0.00247120966252696	0.00553972592594905	0.000146768228148806	0.0000579098345333786

meta cleavage pathway of aromatic compounds	PWY-5430	0.00263456609584351	0.00587806492138669	0.0000151751167164381	0.0000949503225006425
factor 420 biosynthesis	PWY-5198	0.00280773814974955	0.00623502415413866	0.00655052391739586	0.00516205441088917
pyruvate fermentation to isobutanol (engineered)	PWY-7111	0.00315148840044692	0.00696567295986632	2.31381551423029E-07	4.64326610558964E-07
anaerobic aromatic compound degradation (Thauera aromatica)	BENZCOA-PWY	0.00318563497040602	0.00700839693489324	0.00448512462924332	0.00495024954193745
phosphopantothenate biosynthesis I	PANTO-PWY	0.00339147106828181	0.00725866884749907	0.00412561559782429	0.00509123651912486
superpathway of L-aspartate and L-asparagine biosynthesis	ASPASN-PWY	0.00339147106828181	0.00725866884749907	0.00365101517522308	0.00303287483826343
L-methionine biosynthesis I	HOMOSER-METSYN-PWY	0.00339147106828181	0.00725866884749907	0.00031927891271247	0.0000758272645332694
protocatechuate degradation I (meta-cleavage pathway)	P184-PWY	0.00339147106828181	0.00725866884749907	0.0049109886570101	0.0054646893358141
polyisoprenoid biosynthesis (E. coli)	POLYISOPRENSYN-PWY	0.00339147106828181	0.00725866884749907	0.0000317263478035459	0.0000040875963143231
chlorosalicylate degradation	PWY-6107	0.00339147106828181	0.00725866884749907	0.00565408189964885	0.00530876028628289
superpathway of guanosine nucleotides de novo biosynthesis II	PWY-6125	0.00383988000135505	0.00810831803857561	0.00667378161390485	0.00717782245565857
glycolysis III (from glucose)	ANAGLYCOLYSIS-PWY	0.00383988000135505	0.00810831803857561	5.78173296735349E-06	0.000014259904223538

toluene degradation V (aerobic) (via toluene-cis-diol)	PWY-5179	0.00383988000135505	0.00810831803857561	0.00357037035393553	0.00428854521694455
NAD salvage pathway I	PYRIDNUCSAL-PWY	0.0040837137563018	0.00854688764040155	0.000368152318422296	0.000121760365503336
L-histidine degradation II	PWY-5028	0.0040837137563018	0.00854688764040155	0.00654761233789887	0.00747750071294353
urate biosynthesis/inosine 5'-phosphate degradation	PWY-5695	0.00434151851779756	0.009046424048098	0.0050171390524938	0.00558904384048459
6-hydroxymethyl-dihydropterin diphosphate biosynthesis III (Chlamydia)	PWY-7539	0.00461399219184859	0.00957201011729992	0.00469590122014299	0.00409962780046888
galactose degradation I (Leloir pathway)	PWY-6317	0.0050323791136614	0.0103943900469949	2.51504915343216E-12	2.6321529524165E-11
dTDP-6-deoxy-α-D-allose biosynthesis	PWY-7413	0.00520480057042518	0.010568160711701	2.39126003547435E-06	6.38660577585327E-06
D-arabinose degradation III	PWY-5519	0.00520588043515083	0.010568160711701	0.0041163404909898	0.00370574493756299
superpathway of L-lysine, L-threonine and L-methionine biosynthesis I	P4-PWY	0.00520588043515083	0.010568160711701	0.00206747263562178	0.00131494040734045
TCA cycle VII (acetate-producers)	PWY-7254	0.00520588043515083	0.010568160711701	0.00196263163774765	0.00288819314032069
superpathway of UDP-N-acetylglucosamine-derived O-antigen building blocks biosynthesis	PWY-7332	0.00552683564137988	0.0109840052872802	0.00436715185976738	0.00384112649990486
L-lysine biosynthesis I	DAPLYSINESYN-PWY	0.00552683564137988	0.0109840052872802	0.000236726298533631	0.0000591363977718484

ketogluconate metabolism	KETOGLUCONMET-PWY	0.0055268356413798 8	0.010984005287280 2	0.000394475154321 69	0.000685801202271 18
biotin biosynthesis II	PWY-5005	0.0055268356413798 8	0.010984005287280 2	0.004913621314567 9	0.005459648335026 04
GDP-mannose biosynthesis	PWY-5659	0.0055268356413798 8	0.010984005287280 2	0.002848368120092 02	0.003573767725830 43
Kdo transfer to lipid IVA III (Chlamydia)	PWY-6467	0.0056548853126238 4	0.011191467585234 6	6.36551430173179 E-12	3.85322076834987 E-11
superpathway of erythromycin biosynthesis (without sugar biosynthesis)	PWY-6975	0.00622284847119	0.012112787353386 3	0.001067520979861 78	0.000661893528028 138
L-leucine degradation I	LEU-DEG2-PWY	0.00622284847119	0.012112787353386 3	0.004532978772422 78	0.004074117014777 77
superpathway of S-adenosyl-L-methionine biosynthesis	MET-SAM-PWY	0.00622284847119	0.012112787353386 3	0.004079028325147 36	0.003715644109330 75
aspartate superpathway	PWY0-781	0.00622284847119	0.012112787353386 3	0.001612984705313 03	0.000979327178303 137
superpathway of ubiquinol-8 biosynthesis (prokaryotic)	UBISYN-PWY	0.0065996328213927	0.012741331936811 2	0.001370171166222 32	0.000832052906329 567
glucose and glucose-1-phosphate degradation	GLUCOSE1PMETAB-PWY	0.0065996328213927	0.012741331936811 2	0.002035053858334 26	0.001408929787049 24
superpathway of UDP-glucose-derived O-antigen building blocks biosynthesis	PWY-7328	0.0069968079197863 2	0.013237960584235 7	0.003073876825527 12	0.003803087400402 64
biotin biosynthesis I	BIOTIN-BIOSYNTHESIS-PWY	0.0069968079197863 2	0.013237960584235 7	0.001636174682751 26	0.000985281125366 759

ubiquinol-7 biosynthesis (prokaryotic)	PWY-5855	0.00699680791978632	0.0132379605842357	0.00163617468275126	0.000985281125366759
ubiquinol-9 biosynthesis (prokaryotic)	PWY-5856	0.00699680791978632	0.0132379605842357	0.00163617468275126	0.000985281125366759
ubiquinol-10 biosynthesis (prokaryotic)	PWY-5857	0.00699680791978632	0.0132379605842357	0.00163617468275126	0.000985281125366759
ubiquinol-8 biosynthesis (prokaryotic)	PWY-6708	0.00785614836698624	0.014687581729583	0.00042928053730205	0.000105316344867547
glycine betaine degradation I	PWY-3661	0.00785614836698624	0.014687581729583	4.95707006065288E-06	8.06521695039678E-06
D-galacturonate degradation II	PWY-6486	0.00785614836698624	0.014687581729583	0.00427320583267501	0.00473620399413773
thiamin salvage II	PWY-6897	0.0083203092261884	0.0153132539454752	0.000271232003602766	0.0000590624764885813
superpathway of vanillin and vanillate degradation	PWY-6338	0.0083203092261884	0.0153132539454752	0.000271232003602766	0.0000590624764885813
vanillin and vanillate degradation I	PWY-7097	0.0083203092261884	0.0153132539454752	0.00029973119700197	0.0000651011792248229
vanillin and vanillate degradation II	PWY-7098	0.0083203092261884	0.0153132539454752	0.00548869204851263	0.00610224207533807
superpathway of L-serine and glycine biosynthesis I	SER-GLYSYN-PWY	0.00932286775191236	0.0170919242118393	0.00112833677010133	0.00055596237488631
superpathway of (Kdo)2-lipid A biosynthesis	KDO-NAGLIPASYN-PWY	0.00986347712747337	0.0179439410819035	0.000120722394449442	0.0000164475041028527

starch degradation III	PWY-6731	0.0098634771274733 7	0.017943941081903 5	0.001416540420739 15	0.001813873665806 58
glycerol degradation to butanol	PWY-7003	0.0100407217538355	0.018196403791433 6	4.5068627682329E-11	1.21607612640207 E-10
superpathway of atrazine degradation	PWY-5724	0.0104299968962077	0.018704863807517 1	1.67884072650417 E-06	2.62186874022481 E-06
vitamin E biosynthesis (tocopherols)	PWY-1422	0.0104318433642815	0.018704863807517 1	0.003598899618647 91	0.004169650639066 03
NAD biosynthesis I (from aspartate)	PYRIDNUCSYN-PWY	0.0104399239855909	0.018704863807517 1	0	1.68766610408278 E-11
aclacinomycin biosynthesis	PWY-7354	0.0110291666911012	0.019612014454476 9	0.006959096255425 13	0.007768675367864 27
adenosine deoxyribonucleotides de novo biosynthesis II	PWY-7220	0.0110291666911012	0.019612014454476 9	0.006959096255425 13	0.007768675367864 27
guanosine deoxyribonucleotides de novo biosynthesis II	PWY-7222	0.0116566861323972	0.020573181121730 9	0.000079607597171 2189	7.69352637543703 E-06
glucose degradation (oxidative)	DHGLUCONATE-PYR-CAT-PWY	0.0116566861323972	0.020573181121730 9	4.41359101503416 E-06	0.000012260356875 3206
superpathway of CDP-glucose-derived O-antigen building blocks biosynthesis	PWY-5823	0.0130074666079587	0.022703069024223 1	0.000636887179887 407	0.001060472628018 99
superpathway of sulfur oxidation (Acidianus ambivalens)	PWY-5304	0.0130074666079587	0.022703069024223 1	0.005067690537566 52	0.005632000142993 92
6-hydroxymethyl-dihydropterin diphosphate biosynthesis I	PWY-6147	0.0130074666079587	0.022703069024223 1	0.000235203943838 664	0.000330577318135 843

allantoin degradation IV (anaerobic)	PWY0-41	0.0137334043088943	0.0236214554112982	0.00215457263127271	0.00153351207824026
L-lysine biosynthesis II	PWY-2941	0.0137334043088943	0.0236214554112982	0.00447460407976376	0.00386553254336167
S-adenosyl-L-methionine cycle I	PWY-6151	0.0137334043088943	0.0236214554112982	0.00102277993991984	0.00132854472824627
superpathway of Clostridium acetobutylicum acidogenic fermentation	PWY-6590	0.0137334043088943	0.0236214554112982	0.0000651170884122546	0.0000246729013240846
mandelate degradation to acetyl-CoA	PWY-6957	0.0144948923475206	0.0247512060663439	0.000009750945203477	0.0000313117342780283
sucrose biosynthesis III	PWY-7347	0.0144948923475206	0.0247512060663439	0.0000319669651267898	0.0000949339005694245
sucrose biosynthesis I (from photosynthesis)	SUCSYN-PWY	0.0152933710301989	0.0259274713164305	0.00709238835001834	0.00755683603111197
superpathway of phospholipid biosynthesis I (bacteria)	PHOSLIPSYN-PWY	0.0152933710301989	0.0259274713164305	0.00553430554149047	0.00594868775637792
superpathway of L-threonine biosynthesis	THRESYN-PWY	0.0161303221067591	0.0272487227017752	0.00083623790366183	0.00109229931568627
pyruvate fermentation to butanoate	CENTFERM-PWY	0.0170072690620831	0.0284255769129516	0.000296199557242843	0.0000837706315177688
chlorophyllide a biosynthesis II (anaerobic)	PWY-5531	0.0170072690620831	0.0284255769129516	0.00519057868863409	0.00615487470034451
starch degradation V	PWY-6737	0.0170072690620831	0.0284255769129516	0.000296199557242843	0.0000837706315177688

chlorophyllide a biosynthesis III (aerobic, light independent)	PWY-7159	0.0179257773574065	0.0295431801047152	0.0000261810724585547	0.0000503611135983581
superpathway of lipopolysaccharide biosynthesis	LPSSYN-PWY	0.0179257773574065	0.0295431801047152	0.00589102151887457	0.0044729465393986
aerobic respiration I (cytochrome c)	PWY-3781	0.0179257773574065	0.0295431801047152	0.00679590490144799	0.00731440654460214
phosphatidylglycerol biosynthesis I (plastidic)	PWY4FS-7	0.0179257773574065	0.0295431801047152	0.00679590490144799	0.00731440654460214
phosphatidylglycerol biosynthesis II (non-plastidic)	PWY4FS-8	0.0198939507675555	0.0326730510869921	0.00216617207100363	0.00158361478702665
superpathway of β-D-glucuronide and D-glucuronate degradation	GLUCUROCAT-PWY	0.0209469580967318	0.0342834296877306	0.0000517167741813151	0.0000588861149035589
1,5-anhydrofructose degradation	PWY-6992	0.0220450213562389	0.0357150819691223	1.79749253119433E-06	5.95551385308433E-06
dTDP-D-desosamine biosynthesis	PWY-6942	0.0220482112790353	0.0357150819691223	0.00176548921786343	0.00132320188733932
superpathway of hexuronide and hexuronate degradation	GALACT-GLUCUROCAT-PWY	0.0220482112790353	0.0357150819691223	0.0008554968904961	0.000506783469443296
cob(II)yrinate a,c-diamide biosynthesis II (late cobalt incorporation)	PWY-7376	0.0256558807787127	0.041282002967475	3.73508044084929E-07	0.0000128759918697528
superpathway of pyrimidine ribonucleosides degradation	PWY-7209	0.0256594267916229	0.041282002967475	0.00407616044972098	0.00337961093899873
L-tryptophan biosynthesis	TRPSYN-PWY	0.0269718544263195	0.0429551755678421	0.00758943098489238	0.00791262948661403

CDP-diacylglycerol biosynthesis I	PWY-5667	0.0269718544263195	0.0429551755678421	0.00758943098489238	0.00791262948661403
CDP-diacylglycerol biosynthesis II	PWY0-1319	0.0269718544263195	0.0429551755678421	0.00297515782582199	0.00246196677039523
superpathway of sulfate assimilation and cysteine biosynthesis	SULFATE-CYS-PWY	0.0283418326973812	0.0449855263955078	0.00205987615428726	0.00150774321280464
sulfate reduction I (assimilatory)	SO4ASSIM-PWY	0.029771346975724	0.0467835452475663	0.00457433616725365	0.00432156888319165
superpathway of L-methionine biosynthesis (transsulfuration)	PWY-5347	0.029771346975724	0.0467835452475663	0.000022705440704335	0.0000338214430286905
adenosine nucleotides degradation IV	PWY-5532	0.029771346975724	0.0467835452475663	0.00178770024304996	0.00214021235492237
pyridoxal 5'-phosphate biosynthesis I	PYRIDOXSYN-PWY	0.0312624231206811	0.0484823807740399	0.0000131413059934326	1.55594470027178E-06
phospholipases	LIPASYN-PWY	0.0312624231206811	0.0484823807740399	0.0023419132795637	0.0028924002049653
purine nucleobases degradation I (anaerobic)	P164-PWY	0.0312624231206811	0.0484823807740399	0.001357052008411	0.000847905237538174
1,4-dihydroxy-2-naphthoate biosynthesis I	PWY-5837	0.0312624231206811	0.0484823807740399	0.0000935921782874719	0.000122332535114439

Table S1E. Relative abundance of the most significant MetaCyc metabolic pathways in the samples collected in periodontitis sites and healthy sites as assessed by Mann-Whitney U tests with false-discovery-rate correction.

cytokines	genus	Correlation	FDR corrected P value
IL-6	k__Bacteria_p__Actinobacteria_c__Actinobacteria_o__Actinomycetales_f__Microbacteriaceae_g__Salinibacterium	0.781058141470492	0.00391896335277532
IL-6	k__Bacteria_p__Actinobacteria_c__Actinobacteria_o__Actinomycetales_f__Nocardioidaceae_g__Propionicimonas	0.77387261365315	0.00391896335277532
IL-6	k__Bacteria_p__Proteobacteria_c__Alphaproteobacteria_o__Rhizobiales_f__Xanthobacteraceae_g__Ancylobacter	0.781058141470492	0.00391896335277532
TNF_alpha	k__Bacteria_p__Proteobacteria_c__Gammaproteobacteria_o__Pseudomonadales_f__Moraxellaceae_g__Psychrobacter	0.743367104494179	0.0080953860942272
TNF_alpha	k__Bacteria_p__Proteobacteria_c__Gammaproteobacteria_o__Xanthomonadales_f__Xanthomonadaceae_g__Pseudoxanthomonas	0.743367104494178	0.0080953860942272
TNF_alpha	k__Bacteria_p__Firmicutes_c__Bacilli_o__Lactobacillales_f__Carnobacteriaceae_g__Carnobacterium	0.704471475170114	0.0260357377017787
IL-6	k__Bacteria_p__Fusobacteria_c__Fusobacteriia_o__Fusobacteriales_f___g__	0.682403924137123	0.0438746482692848

Table S2A. Most significant correlations between cytokines and oral microbiome collapsed at genus level in the samples collected in healthy sites as assessed by Pearson correlation with false-discovery-rate correction.

cytokines	genus	Correlation	FDR corrected P value
IL-4	k__Bacteria_p__Actinobacteria_c__Actinobacteria_o__Actinomycetales_f__Actinomycetaceae_g__	0.913181895820066	2.80808337152153e-07
IL-4	k__Bacteria_p__Actinobacteria_c__Coriobacteriia_o__Coriobacteriales_f__Coriobacteriaceae_g__Atopobium	0.917147889369325	2.80808337152153e-07
IL-4	k__Bacteria_p__Firmicutes_c__Clostridia_o__Clostridiales_f__Lachnospiraceae_g__Lachnoanaerobaculum	0.866304124952008	1.72575647575241e-05

IL-4	k__Bacteria_p__Proteobacteria_c__Alphaproteobacteria_o__Rhizobiales_f__Bradyrhizobiaceae___	0.840219451283567	8.09320296113803e-05
IL-10	k__Bacteria_p__Actinobacteria_c__Actinobacteria_o__Actinomycetales_f__Propionibacteriaceae_g__Propionibacterium	0.799843625509915	0.000631656205793434
IL-10	k__Bacteria_p__Firmicutes_c__Bacilli_o__Bacillales_f__Staphylococcaceae_g__Staphylococcus	0.788520300439248	0.000910949715131011
IL-10	k__Bacteria_p__Actinobacteria_c__Actinobacteria_o__Actinomycetales_f__Cellulomonadaceae_g__Actinotalea	0.782716791377285	0.00102134432047806
IL-4	k__Bacteria_p__Actinobacteria_c__Actinobacteria_o__Actinomycetales_f__Propionibacteriaceae_g__	0.708536604395631	0.0154468691733736
IL-6	k__Bacteria_p__Proteobacteria_c__Alphaproteobacteria_o__Rhodobacterales_f__Rhodobacteraceae___	0.696710762528417	0.019988306088363
TNF_alpha	k__Bacteria_p__Actinobacteria_c__Actinobacteria_o__Actinomycetales_f__Actinomycetaceae_g__Actinomyces	0.668454807635251	0.0277109389384337
TNF_alpha	k__Bacteria_p__Firmicutes_c__Bacilli_o__Lactobacillales_f__Carnobacteriaceae_g__	0.663662633943241	0.0277109389384337
TNF_alpha	k__Bacteria_p__Firmicutes_c__Clostridia_o__Clostridiales_f__Clostridiaceae___	0.671428752259426	0.0277109389384337
TNF_alpha	k__Bacteria_p__Firmicutes_c__Clostridia_o__Clostridiales_f__Lachnospiraceae_g__Clostridium	0.664095655949535	0.0277109389384337
IL-17A	k__Bacteria_p__Firmicutes_c__Clostridia_o__Clostridiales_f__Veillonellaceae_g__Acidaminococcus	0.67309449622526	0.0277109389384337
TNF_alpha	k__Bacteria_p__Fusobacteria_c__Fusobacteriia_o__Fusobacteriales_f__Fusobacteriaceae_g__Fusobacterium	0.664333340860748	0.0277109389384337
IL-6	k__Bacteria_p__Proteobacteria_c__Betaproteobacteria_o__Neisseriales_f__Neisseriaceae___	0.66389043248005	0.0277109389384337
TNF_alpha	k__Bacteria_p__Proteobacteria_c__Betaproteobacteria_o__Rhodocyclales_f__Rhodocyclaceae_g__Propionivibrio	0.679586299145746	0.0277109389384337
TNF_alpha	k__Bacteria_p__Bacteroidetes_c__Sphingobacteriia_o__Sphingobacteriales_f__Sphingobacteriaceae_g__Sphingobacterium	0.655905782117121	0.0322653625660387
TNF_alpha	k__Bacteria_p__Proteobacteria_c__Alphaproteobacteria_o__Rhodobacterales_f__Rhodobacteraceae_g__Amaricoccus	0.646276811080073	0.0373568106369682

IL-17A	k__Bacteria_p__Proteobacteria_c__Betaproteobacteria_o__Rhodocyclales_f__Rhodocyclaceae_g__Propionivibrio	0.647084788419367	0.0373568106369682
TNF_alpha	k__Bacteria_p__Actinobacteria_c__Actinobacteria_o__Actinomycetales_f__Microbacteriaceae_g__Microbacterium	0.639484638392383	0.041842599521803
IL-17A	k__Bacteria_p__Proteobacteria_c__Betaproteobacteria_o__Neisseriales_f__Neisseriaceae___	0.638043099536067	0.041842599521803

Table S2B. Most significant correlations between cytokines and oral microbiome collapsed at genus level in the samples collected in periodontitis sites as assessed by Pearson correlation with false-discovery-rate correction.

**cytokines**	**PATHWAYS**	**Correlation**	**FDR corrected P value**
IL-6	PWY-1882 superpathway of C1 compounds oxidation to CO2	0.778140117786349	0.0215331440046045
IL-10	PWY-1861 formaldehyde assimilation II (RuMP Cycle)	-0.74049363300693	0.0498192403823827
IL-10	RUMP-PWY formaldehyde oxidation I	-0.725846352984813	0.0563313324492285

Table S2C. Most significant correlations between cytokines and predicted metagenomic functions (KEGG pathways) in the samples collected in healthy sites as assessed by Pearson correlation with false-discovery-rate correction.

**cytokines**	**PATHWAYS**	**Correlation**	**FDR corrected P value**
IL-17A	BENZCOA-PWY anaerobic aromatic compound degradation (Thauera aromatica)	0.752822764264593	0.0606265156466369
IL-6	PWY-5499 vitamin B6 degradation	0.735332831719876	0.0606265156466369
IL-6	PWY-7402: benzoate fermentation (to acetate and cyclohexane carboxylate)	benzoate fermentation (to acetate and cyclohexane carboxylate)	0.0622478814555644

IL-17A	DENITRIFICATION-PWY nitrate reduction I (denitrification)	0.704120083027345	0.0702784389741
IL-17A	PWY-7402 benzoate fermentation (to acetate and cyclohexane carboxylate)	0.705471289009918	0.0702784389741

Table S2D. Most significant correlations between cytokines and predicted metagenomic functions (KEGG pathways) in the samples collected in periodontitis sites as assessed by Pearson correlation with false-discovery-rate correction.

ENZYMES	ENZYMES names	cytokines	Correlation	FDR corrected P value
1.1.2.7	Methanol dehydrogenase (cytochrome c)	IL-6	0.773298386832442	0.0602654641172393
1.14.19.4	Delta(8)-fatty-acid desaturase	IL-6	0.759263905392162	0.0602654641172393
3.4.23.49	Omptin	IL-6	0.779864140905324	0.0602654641172393
4.1.2.55	aldolase	IL-6	0.758808375620779	0.0602654641172393
4.2.1.66	Cyanide hydratase	IL-6	0.759263905392162	0.0602654641172393

Table S2E. Most significant correlations between cytokines and predicted metagenomic functions (Enzyme Commission) in the samples collected in healthy sites as assessed by Pearson correlation with false-discovery-rate correction.

ENZYMES	ENZYMES names	cytokines	Correlation	FDR corrected P value
1.1.1.105	All-trans-retinol dehydrogenase (NAD(+))	IL-4	0.869243233182877	0.000310357115539233
2.3.2.17	glycyltransferase	IL-4	0.875337744439791	0.000310357115539233
3.5.3.25	N(omega)-hydroxy-L-arginine amidinohydrolase	IL-4	0.83548087667382	0.0021944621149222
1.14.16.1	Phenylalanine 4-monooxygenase	IL-17A	0.826476037589159	0.00282911021140912
1.3.1.75	Divinyl chlorophyllide a 8-vinyl-reductase	IL-6	0.797171936216008	0.0108841160838587
1.14.13.9	Kynurenine 3-monooxygenase	TNF_alpha	0.777746252700735	0.0182933718653553
1.3.8.2	4,4'-diapophytoene desaturase (4,4'-diapolycopene-forming)	IL-10	0.768546994701519	0.0182933718653553

1.8.1.14	CoA-disulfide reductase	IL-10	0.768546993690475	0.0182933718653553
2.3.2.18	glycyltransferase	IL-4	0.773350766173424	0.0182933718653553
2.5.1.96	4,4'-diapophytoene synthase	IL-10	0.768546994552137	0.0182933718653553
3.1.27.6	Enterobacter ribonuclease	IL-6	0.778650981191651	0.0182933718653553
1.10.2.2	Quinol--cytochrome-c reductase	IL-17A	0.75088023364689	0.0242933073163435
1.5.1.36	Flavin reductase (NADH)	IL-6	0.753389316525921	0.0242933073163435
1.7.2.4	Nitrous-oxide reductase	IL-17A	0.751903404506183	0.0242933073163435
2.3.1.207	Beta-ketodecanoyl-[acyl-carrier-protein] synthase	TNF_alpha	0.756946660085144	0.0242933073163435
2.8.3.21	L-carnitine CoA-transferase	IL-4	0.75236649952096	0.0242933073163435
4.2.1.12	Phosphogluconate dehydratase	IL-6	0.752089958274913	0.0242933073163435
3.4.21.72	IgA-specific serine endopeptidase	IL-6	0.74458192422656	0.0292351909194497
1.17.1.1	CDP-4-dehydro-6-deoxyglucose reductase	IL-6	0.734277826214766	0.0405800309771708
1.2.1.68	Coniferyl-aldehyde dehydrogenase	TNF_alpha	0.725785181933035	0.0521493291506078
2.3.2.5	Glutaminyl-peptide cyclotransferase	TNF_alpha	0.718330708180412	0.0599002396009973
2.4.1.5	Dextransucrase	TNF_alpha	0.717679891195155	0.0599002396009973
4.2.1.53	Oleate hydratase	TNF_alpha	0.718342687694091	0.0599002396009973
4.2.1.57	Isohexenylglutaconyl-CoA hydratase	TNF_alpha	0.711890489569407	0.0696334110066253
1.1.5.8	Quinate dehydrogenase (quinone)	TNF_alpha	0.703355626100141	0.0816112171008318
3.3.2.1	Isochorismatase	TNF_alpha	0.704536885969368	0.0816112171008318
3.5.1.96	Succinylglutamate desuccinylase	TNF_alpha	0.703558895079998	0.0816112171008318
2.1.1.67	Thiopurine S-methyltransferase	TNF_alpha	0.700895777046166	0.0850759035649384
1.1.3.12	Pyridoxine 4-oxidase	IL-6	0.694091396743781	0.0905060814470634
1.14.13.131	Dimethyl-sulfide monooxygenase	IL-6	0.694688764899713	0.0905060814470634
1.2.1.72	Erythrose-4-phosphate dehydrogenase	TNF_alpha	0.695793119570367	0.0905060814470634
2.1.3.1	Methylmalonyl-CoA carboxytransferase	IL-6	0.692642965310999	0.0905060814470634

5.3.1.26	Galactose-6-phosphate isomerase	IL-6	0.696249620980044	0.0905060814470634
5.3.3.8	Dodecenoyl-CoA isomerase	TNF_alpha	0.692893829833396	0.0905060814470634
1.1.1.67	Mannitol 2-dehydrogenase	TNF_alpha	0.688250398965159	0.0925809501551899
1.14.11.19	Leucocyanidin oxygenase	TNF_alpha	0.673481630066119	0.0925809501551899
1.14.12.14	2-aminobenzenesulfonate 2,3-dioxygenase	TNF_alpha	0.673481630066139	0.0925809501551899
1.14.15.9	Spheroidene monooxygenase	IL-6	0.68784471866614	0.0925809501551899
1.16.1.3	Aquacobalamin reductase	TNF_alpha	0.687012423909958	0.0925809501551899
1.17.5.1	Phenylacetyl-CoA dehydrogenase	TNF_alpha	0.673461370768913	0.0925809501551899
1.18.1.6	Adrenodoxin-NADP(+) reductase	TNF_alpha	0.673482137546604	0.0925809501551899
1.3.1.19	Cis-1,2-dihydrobenzene-1,2-diol dehydrogenase	TNF_alpha	0.681887070958801	0.0925809501551899
1.3.1.62	Pimeloyl-CoA dehydrogenase	TNF_alpha	0.674271020293024	0.0925809501551899
1.4.9.2	Aralkylamine dehydrogenase (azurin)	TNF_alpha	0.673480929371479	0.0925809501551899
1.5.1.37	FAD reductase (NADH)	TNF_alpha	0.673481620408504	0.0925809501551899
1.5.1.41	Riboflavin reductase (NAD(P)H)	TNF_alpha	0.687012423909958	0.0925809501551899
2.1.1.174	23S rRNA (guanine(1835)-N(2))-methyltransferase	TNF_alpha	0.68424502819233	0.0925809501551899
2.1.1.187	23S rRNA (guanine(745)-N(1))-methyltransferase	TNF_alpha	0.685403930557047	0.0925809501551899
2.3.1.230	2-heptyl-4(1H)-quinolone synthase	TNF_alpha	0.674479269437082	0.0925809501551899
2.5.1.39	4-hydroxybenzoate polyprenyltransferase	IL-6	0.680331190099643	0.0925809501551899
2.7.1.89	Thiamine kinase	TNF_alpha	0.680626041302477	0.0925809501551899
2.7.11.5	[Isocitrate dehydrogenase (NADP(+))] kinase	TNF_alpha	0.682515078565384	0.0925809501551899
2.7.7.58	(2,3-dihydroxybenzoyl)adenylate synthase	TNF_alpha	0.688588475577676	0.0925809501551899
3.1.1.56	Methylumbelliferyl-acetate deacetylase	TNF_alpha	0.67313064148987	0.0925809501551899
3.1.1.84	Cocaine esterase	TNF_alpha	0.67313064148987	0.0925809501551899
3.1.2.21	Dodecanoyl-[acyl-carrier-protein] hydrolase	TNF_alpha	0.680514697309054	0.0925809501551899
3.1.2.25	Phenylacetyl-CoA hydrolase	TNF_alpha	0.673461370768913	0.0925809501551899

3.11.1.3	Phosphonopyruvate hydrolase	TNF_alpha	0.67348150156595	0.0925809501551899
4.1.3.45	3-hydroxybenzoate synthase	TNF_alpha	0.677704069620328	0.0925809501551899
3.6.3.33	Vitamin B12-transporting ATPase	TNF_alpha	0.672457923991846	0.0927878998168351
2.6.1.87	UDP-4-amino-4-deoxy-L-arabinose aminotransferase	TNF_alpha	0.670344055081281	0.0937888980299378
2.7.1.13	Dehydrogluconokinase	TNF_alpha	0.670492432188585	0.0937888980299378
2.7.11.26	[Tau protein] kinase	TNF_alpha	0.671428167068617	0.0937888980299378
2.7.1.73	Inosine kinase	TNF_alpha	0.669243977655225	0.0939098900666312
3.5.1.38	Glutamin-(asparagin-)ase	TNF_alpha	0.669181301430355	0.0939098900666312
2.7.1.8	Glucosamine kinase	TNF_alpha	0.66778349651648	0.0955178620089822
4.2.2.3	Poly(beta-D-mannuronate) lyase	TNF_alpha	0.667482893520241	0.0955178620089822
1.1.1.43	Phosphogluconate 2-dehydrogenase	TNF_alpha	0.664517950891738	0.099221005104641
3.1.1.74	Cutinase	IL-10	0.665054152486899	0.099221005104641
3.4.24.20	Peptidyl-Lys metalloendopeptidase	TNF_alpha	0.664636714155371	0.099221005104641

Table S2F. Most significant correlations between cytokines and predicted metagenomic functions (Enzyme Commission) in the samples collected in periodontitis sites as assessed by Pearson correlation with false-discovery-rate correction.
